# A systematic review of racial/ethnic and socioeconomic disparities in COVID-19

**DOI:** 10.1186/s12939-021-01582-4

**Published:** 2021-11-24

**Authors:** Ahmad Khanijahani, Shabnam Iezadi, Kamal Gholipour, Saber Azami-Aghdash, Deniz Naghibi

**Affiliations:** 1 Department of Health Administration and Public Health, John G. Rangos School of Health Sciences, Duquesne University, Pittsburgh, PA USA; 2 Hospital Management Research Center, Iran University of Medical Sciences, Tehran, Iran; 3 Social Determinants of Health Research Center, Department of Health Service Management, School of Management and Medical Informatics, Tabriz University of Medical Sciences, Tabriz, Iran; 4 Tabriz Health Services Management Research Center, Health Management and Safety Promotion Research Institute, Tabriz University of Medical Sciences, Tabriz, Iran; 5 Student Research Committee, Tabriz University of Medical Sciences, Tabriz, Iran

**Keywords:** COVID-19, Disparities, Socioeconomic status, Race, Ethnicity, Vulnerable population

## Abstract

**Background:**

Preliminary evidence from the COVID-19 pandemic shows the presence of health disparities, especially in terms of morbidity and mortality. This study aimed to systematically review the evidence on the association of racial/ethnic and socioeconomic status (SES) with health outcomes and access to healthcare services during the COVID-19 pandemic.

**Methods:**

We retrieved published evidence from late December 2019 through March 1, 2021. The target population was the population of the countries during the COVID-19 pandemic. The exposures were defined as belonging to racial/ethnic minority groups and/or low SES. The primary outcomes of interest include (1) death from COVID-19, (2) COVID-19 incidence/infection, (3) COVID-19 hospitalization, (4) ICU admission, (5) need for mechanical ventilation, (6) confirmed diagnosis, and (7) access to testing. We systematically synthesized the findings from different studies and provided a narrative explanation of the results.

**Results:**

After removing the duplicate results and screening for relevant titles and abstracts, 77 studies were selected for full-text review. Finally, 52 studies were included in the review. The majority of the studies were from the United States (37 studies). Despite the significant incongruity among the studies, most of them showed that racial/ethnic minority groups had higher risks of COVID-19 infection and hospitalization, confirmed diagnosis, and death. Additionally, most of the studies cited factors such as low level of education, poverty, poor housing conditions, low household income, speaking in a language other than the national language in a country, and living in overcrowded households as risk factors of COVID-19 incidence/infection, death, and confirmed diagnosis. However, findings in terms of the association of lack of health insurance coverage and unemployment with the outcome measures as well as the association of requiring mechanical ventilation, ICU admission, and access to testing for COVID-19 with race/ethnicity were limited and inconsistent.

**Conclusion:**

It is evident that racial/ethnic minority groups and those from low SES are more vulnerable to COVID-19; therefore, public health policymakers, practitioners, and clinicians should be aware of these inequalities and strive to narrow the gap by focusing on vulnerable populations. This systematic review also revealed a major incongruity in the definition of the racial/ethnic minority groups and SES among the studies.

**Systematic review registration:**

PROSPERO CRD42020190105.

**Supplementary Information:**

The online version contains supplementary material available at 10.1186/s12939-021-01582-4.

## Background

Since its first reported case in late December 2019 in Wuhan, China, and rapid global spread, Coronavirus Disease 2019 (COVID-19) has resulted in devastating consequences [1, 2] and was declared as a global public health emergency on March 11, 2020, by the World Health Organization (WHO) [3]. Due to the absence of confirmed treatment and vaccination for about 12 months after the onset of the outbreak, most of the research was focused on clinical, pharmaceutical, and epidemiological aspects of the pandemic [3, 4]. Nevertheless, this does not downplay the role of public health in ensuring equity in access to needed services [5].

Racial/ethnic and Socioeconomic Status (SES) disparities in health are prominent [6–8] and have been linked directly or indirectly to individual life expectancy and mortality [9]. Evidence shows a connection between lower SES and higher risk of infectious diseases’ incidence and severity [10–12]. Therefore, narrowing the disparity gaps or eradicating racial/ethnic and SES disparities is a public health priority [13]. This calls for research studies to identify and understand the status of disparities across different population groups [7, 14]. Because their specific SES can potentially influence the incidence and severity of the disease in various ways, populations of low-SES and racial/ethnic minority groups should be considered as high-risk populations during the epidemics [15].

Racial/ethnic and SES disparities are observed in the health care utilization and health outcomes of populations during the COVID-19 pandemic, especially in terms of morbidity and mortality [5, 16, 17]. Gross disparities in hospitalization rates and mortality between racial/ethnic groups in the context of COVID-19 highlight the shortcomings of public health strategies in achieving “optimal health for all” [5, 18]. For instance, several studies have shown disproportionate adverse effects of COVID-19 on African Americans [16, 19–21]. Disparities in COVID-19 is not merely a concern of developed countries. It may affect Low- and Middle-Income Countries (LMICs) even more severely [22–24]. Nevertheless, many LMICs do not have appropriate surveillance systems and responsive healthcare infrastructures [25] to address such issues reliably.

It is well-documented that individuals from low-SES or racial/ethnic minority groups are more vulnerable to COVID-19 [12, 26, 27]. It is crucial to explore the racial/ethnic and SES disparities to reach a precise understanding of the presence and extent of potential disparities in the context of COVID-19. Additionally, several commentaries and editorials have highlighted the essential need for exploring the disparities in COVID-19 to conduct early interventions [5, 28–30]. Although some studies suggest the existence of racial/ethnic and SES disparities in COVID-19, the evidence is inconsistent. For example, while some studies reported higher mortality rates in racial/ethnic minority groups [12, 31, 32], some other studies did not report such differences by race/ethnicity [33, 34]. Conducting a review study to systematically collect data and synthesize the findings from different studies may provide researchers and policymakers with insights into possible disparities in COVID-19.

Although a review study has rigorously examined the disparities in the era of COVID-19, this study has focused only on clinical outcomes with a narrower focus on race/ethnicity [30]. We have included a broader range of outcomes in the racial/ethnic minority groups and low SES populations. Two main objectives of this study include (1) to systematically review the evidence on the association of race/ethnicity with health outcomes and health resources in the context of COVID-19, and (2) to systematically review the evidence on the association of SES with health outcomes and healthcare resources in the context of COVID-19.

## Methods

This systematic review adheres to the four-step flowchart suggested by the Preferred Reporting Items for Systematic Reviews and Meta-Analyses (PRISMA) Statement [35].

### Eligibility criteria for the inclusion of studies

#### Population

The target population includes the population of countries during the pandemic of COVID-19.

#### Exposure

**Fig. 1:**
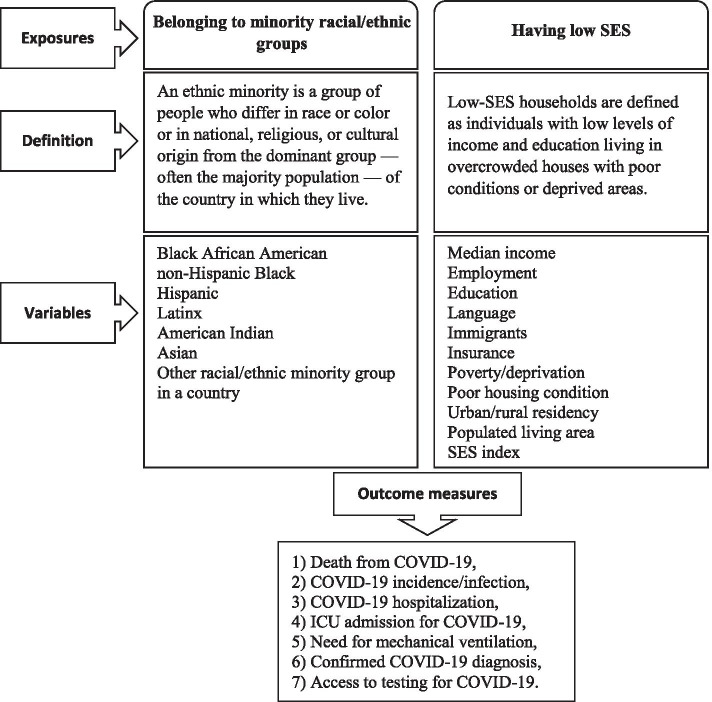
Characteristics and definition of the exposures and types of the outcome measures

The exposure is defined as belonging to racial/ethnic minority and/or low-SES populations. A framework showing the types and definitions of the exposures is presented in Fig. 1.

#### Outcomes

The main outcomes of interest include 1) death from COVID-19, 2) COVID-19 incidence/infection, 3) COVID-19 hospitalization, 4) ICU admission for COVID-19, 5) need for mechanical ventilation, 6) confirmed diagnosis for COVID-19, and 7) access to testing for COVID-19.

#### Types of studies

We included empirical English-language peer-reviewed observational studies in the review. We excluded the non-peer-reviewed papers, editorials, reviews, commentaries, mathematical modeling reports, and methodological and conceptual papers from the review.

### Search strategy and information sources

Keywords were identified in three main domains—coronavirus infectious disease, disparity, and racial/ethnic minority groups—through a rapid literature review and using medical subject heading (MeSh) and Embase subject headings (EMTREE) (Table 1). An experienced medical librarian contributed to the development of the preliminary search strategy. In the first step, we searched each of the domains independently, followed by a comprehensive search using a composition of all domains to ensure that the final search strategy was appropriate. Complete search strategies are provided in the supplementary file (Additional file 1 in supplementary files)

**Table 1:** key terms for database search strategy

Concept 1: Coronavirus Disease 2019	Concept 2: Outcomes/Access	Concept 3: Race, Ethnicity, SES
COVID-19 OR COVID19	Access	Ethnicity OR Disparity
SARS-CoV-2	Mortality	Race OR Minority
Coronavirus 2019	Morbidity	Inequity
2019-nCoV OR 2019ncov	Hospitalization	Socioeconomic
coronavirus disease-19	Utilization	Poverty
Search strategy: 1 AND 2 AND 3		

We searched major electronic databases (from late 2019 onwards), including Medline (via PubMed), Web of Science, Cochrane Library, Scopus, CINAHL, and Embase, on November 20, 2020, and updated on March 1, 2021. Moreover, we searched the references and citation lists of relevant studies, Google Scholar search engine, preprint sources such as medRxiv, WHO website, and other relevant sources to identify the relevant studies.

### Data management and extraction

After pooling all search results in the Endnote reference management software program and removing duplicated results, two reviewers screened the titles/abstracts to find all relevant studies (KG and SA). Then, two researchers carefully read the full text of the relevant studies based on the eligibility criteria (SI and AK). Three authors (SI, AK, and DN) extracted essential information and entered it into the data extraction table designed in Microsoft Word. The extraction form contained items such as bibliographic information, type of study, participants and sample size, race/ethnicity ascertainment source, study settings, data source, exposure and outcomes, and main results (See Additional file 2 in supplementary files). The investigators conducted a pilot test of three articles to ensure the validity of the data extraction form before starting the main data extraction.

### Assessment of methodological quality

Using standardized critical appraisal instruments for observational studies from Joanna Briggs Institute (JBI) [36], two investigators (SA and DN) independently assessed the quality of eligible studies. This measure was taken to confirm the internal validity of the review’s findings and prevent problems of spurious precision due to confounded or biased statistics. The studies were divided into three categories: cross-sectional studies, cohort studies, and case-control studies, and relevant checklists were used to assess each study type. The evaluation checklists included 8, 11, and 10 questions for cross-sectional, cohort, and case-control studies, respectively. The answers to each item in these tools were “Yes”, “No”, “Unclear” or “Not applicable”. The answer “yes” was assigned the score “1” and answers “no”, “cannot be answered,” or “not applicable” were scored “0”. The quality score of each study was calculated and reported as a percentage. The final score for each study was given by agreement between the two evaluators. Any disagreement among the two investigators was addressed and solved by discussing it with the other authors.

### Data synthesis

Due to the high level of heterogeneity in the study settings, participants, and methods, as well as different types of exposures and outcome measures, we did not conduct Meta-analysis. By aggregating the information extracted from each manuscript, we synthesized and organized the results from the included studies. We also explained the main results in narrative and tabular formats.

## Results

**Fig. 2:**
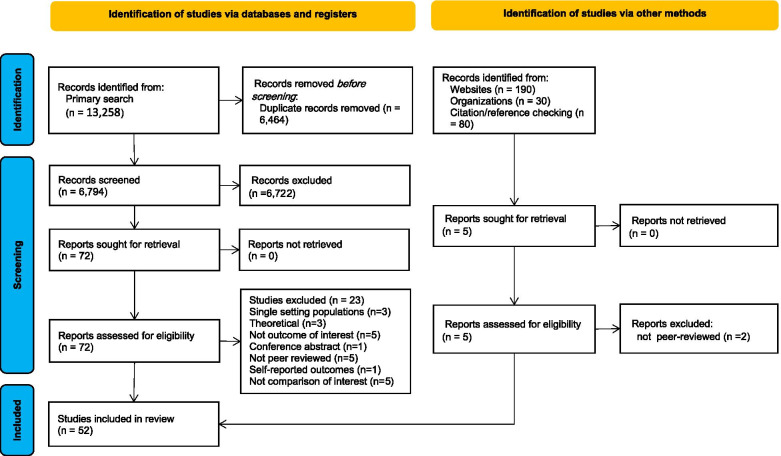
Prisma diagram for study selection

Our search in major electronic databases yielded 13,558 results. After removing the repetitive results and screening for relevant title/abstracts, 77 studies were selected for full-text review. Finally, 52 studies were included in the review [22–24, 26, 27, 32–34, 37–80]. The study selection process and reasons for excluding the studies after full-text review are presented in Fig. 2.

The quality assessment results of the included studies showed that the average score of cross-sectional studies was 86.25%, cohort studies was 90.9%, and case-control studies was 100%. Among the cross-sectional studies, one of the most problematic items was strategies to deal with confounding factors stated. Among cohort studies, strategies to address incomplete follow-up was the main problem (See Additional file 3 in the supplementary files).

The majority of the studies were from the United States (37 studies). Studies have targeted different race/ethnicity and SES characteristics to examine the disparities in COVID-19 outcomes. Almost all studies used self-reported data on race/ethnicity, either already documented in the database they used for data collection, or gathered and recorded in the patients’ medical documents. A total of 29 out of 52 studies examined the disparities using individual-level data.

### Racial/ethnic disparities and COVID-19 outcomes

A total of 40 studies examined the association between race/ethnicity and COVID-19 outcomes, including the risk of incidence/infection, confirmed diagnosis, access to testing, hospitalization, ICU admission, death, and need for mechanical ventilation. According to the results of 11 studies [32, 33, 37, 43, 47, 55, 74, 75, 77, 79, 80] some disadvantaged racial/ethnic minority groups were at a higher risk of COVID-19 infection compared to their White counterparts or those from racial/ethnic majority groups in the region. In contrast, one study from the US did not report a significant association between a higher proportion of Black or Asian residents and a higher risk of COVID-19 incidence/infection [68].

Concerning COVID-19 deaths, nine studies [33, 42, 44, 48, 57, 61, 64, 65, 68] did not report significant associations between race/ethnicity and risk of COVID-19 death; 11 studies [23, 26, 32, 39, 46, 47, 55, 59, 69, 74, 79, 80] reported that racial/ethnic minority groups were at greater risk of dying from COVID-19. However, in contrast, two studies showed reverse association [49, 50]. There was also incongruity in the results of other studies [37, 41, 60, 62, 63, 66]. For example, a study in the US showed a statistically significantly increased hazard of in-hospital mortality for Hispanics and Asians. Nevertheless, there was no significant increased risk of in-hospital mortality for African Americans compared to Whites [60]. Another study from the US also concluded that Hispanics were two times more likely to die from COVID-19 than Whites, while there was a nonsignificant difference comparing Blacks and Whites [41]. According to another study from the US, compared with non-Hispanic White patients, Black patients and Hispanic patients were at a lower risk of death from COVID-19, whereas Asian patients had a higher risk of mortality [62]. Another study showed that although the US population of South Asians was at a higher risk of COVID-19 death compared with the rest of the population, there was no significantly higher risk of COVID-19 death for Blacks [63]. One study from the United Kingdom showed that whereas the risk of COVID-19 death was not statistically significantly different between Black or Mixed/Other racial/ethnic populations compared to Whites, Asians showed a higher risk of COVID-19 death than Whites [66]. A study from the US reported that while African American population was at a higher risk of death from COVID-19, this was not the case for Asians [37].

Concerning confirmed diagnosis, ten studies [27, 33, 41, 48, 50, 57, 58, 61, 67, 72] showed that racial/ethnic minority groups were more likely to have positive test results than the rest of the population tested for COVID-19. However, some studies reported inconsistent results for different racial/ethnic groups. For example, a study from the US showed a statistically significant association between the positive COVID-19 test results and belonging to Black, Hispanic, or Asian racial/ethnic populations. However, this association was reversed for the non-Hispanic Asian population living in the high-density populated areas [51]. On the other hand, one study showed that while the Latinx ethnic group had a higher likelihood of positive test results than non-Latinx patients, Black patients had fewer positive test results than non-Black patients [56]. See Table 2 for more information.

**Table 2:** Summary of the results of included studies

Author/ year	Type of study; Country; setting	Participants/ Sample size	Exposure	Outcome measures	Results
(Abedi et al., 2020) [37]	Ecological study; US; 369 counties from seven most affected states.	*N* = 369 counties with 102,178,117 population	Black; Asian; Hispanic; Insurance; Poverty; Income; Education.	COVID-19 infection; COVID-19 death.	▪ Counties with a higher median income had a higher rate of infection. ▪ A higher percentage of Asians, or Blacks, or Hispanics were associated with a higher rate of infection while a higher percentage of non-Hispanic Whites was associated with a lower rate of COVID-19. ▪ There were no significant associations between percentage of uninsured and risk of COVID-19 infection. ▪ Higher percentage of people under the poverty level (for all the races analyzed in this study), or a higher percentage of people on Medicaid were significantly associated with higher mortality in the counties. ▪ African American population was at higher risk of mortality from COVID-19, conversely, Asians were not.
(Adegunsoye et al., 2020) [33]	Retrospective cohort study; US; University of Chicago.	*N* = 4413 patients tested for COVID-19 from January 1 to April, 15, 2020. Black (58%) White (24%) Other (18%)	Black	Covid-19 infection; Covid-19 death; Hospitalization; Confirmed diagnosis.	▪ Risk of Covid-19 infection in Black race was 3.3 times higher compared to whites. ▪ Risk of Covid-19 hospitalization was 3.8 times higher for Black race compared to whites. ▪ Age-adjusted SARS-CoV-2 positivity rate (0.14) remained higher in Black individuals compared with non-Black individuals (0.19 vs. 0.07). ▪ There were no observed racial differences in mortality among all SARS-CoV-2–positive patients in the entire cohort.
(Adhikari et al., 2020) [32]	Cross-sectional study; US; Urban US counties at 10 major us cities with early surges of covid-19 infections.	*N* = 158 counties	Minority (not specified): categorizing counties as Substantially White, Less diverse, More diverse, and Substantially non-White;	Cumulative incidence (per 100,000); COVID-19 death (per 100,000).	▪ In more-poverty counties, those with substantially non-White populations had an infection rate 7.8 times that of counties with substantially White populations and had a death rate 9.3 times greater. ▪ Among both more-poverty and less-poverty counties, those with substantially non-White or more diverse populations had higher expected cumulative COVID-19 incident infections compared with counties with substantially White or less-diverse populations. ▪ Among both more-poverty and less-poverty counties, those with substantially non-White or more diverse populations had higher observed COVID-19 deaths rate.
(Ahmad et al., 2020) [38]	cross sectional ecological study; US; 3135 US counties.	*N* = 3135 US counties	Percentage of households in a county with poor housing condition	Incidence rate ratios (per 100,000); Mortality rate ratios (per 100,000);	▪ In the adjusted models standardized by county population, with each 5% increase in percent households with poor housing conditions, there was a 50% higher risk of COVID-19 incidence and a 42% higher risk of COVID-19 mortality.
(Aldridge et al., 2020) [39]	Prospective cohort study; England	*N* = 16,272 deaths from COVID-19 Asian (8%) Black (6%) Mixed, other (2%) White (81%)	Minority ethnic group: Black African, Black Caribbean, Pakistani, Bangladeshi and Indian.	COVID-19 death (standardized mortality ratios (SMRs)	▪ Adjusting for region, there was a lower risk of death for White Irish and White British ethnic groups, but increased risk of death for Black African, Black Caribbean, Pakistani, Bangladeshi and Indian minority ethnic groups.
(Azar et al., 2020) [34]	Retrospective cohort study; US; Sutter Health, a large not-for-profit integrated health system in northern California.	Group1 *N* = 14,036 patients who were tested for COVID-19 from January 1–April 8, 2020 Group2 *N* = 1052 confirmed cases of COVID-19 By April 8 Non-Hispanic White (89%) Asian (22%) African American (12.5%) American Indian/Pacific Islander (0.9%) Hispanic (44.9%) Other (30.8%)	African American; Household income (median income level by Census ZIP Code Tabulation Areas (ZCTAs) categorized by quartiles); Insurance.	COVID-19 testing; COVID-19 hospitalization	▪ A smaller percentage of African Americans (29.9%) were tested for COVID-19 in an ambulatory setting compared to whites (56.0%), Asians (60.0%), and Hispanics (53.8%) (exhibit 2). The majority of African Americans were tested in hospitals, either in the emergency department (37.8%) or as inpatients (32.3%). ▪ The likelihood of hospital admission for African Americans was 2.7 times higher than that of whites. ▪ People with Medicaid or who were self-pay or had no reported insurance had twice the odds of being admitted, compared to those with commercial insurance. ▪ COVID-19 positive patients residing in ZIP codes within the top two quartiles of income (quartiles 3 and 4) were less likely to be admitted to the hospital than those residing in the bottom quartile ZIP code (OR = 0.24 and 0.55 for the top two quartiles).
(Baqui et al., 2020) [22]	Cross-sectional observational study; Brazil; Central-south region (Cs) and north region (n)	*N* = 11,321 patients withCOVID-19 White (27.8% n; 58.7% Cs) Pardo (61.5% n; 33.2% Cs) Black (8.8% n; 6.8% Cs) East Asian (1.2% n; 1.1% Cs) Indigenous (0.7% n; 0.3% Cs)	Black	COVID-19 deaths	▪ Compared with White Brazilians, Pardo Brazilians had 45% and Black Brazilians had 58% higher risk of mortality for Covid-19.
(de Lusignan et al., 2020) [27]	Cross-sectional study; UK	*N* = 3802 SARS-CoV-2 test results White (65.7%) Asian (4.0%) Black (1.5%) Mixed, other, unknown (28.8%)	Black; Urban; Deprivation.	Confirmed diagnosis	▪ Compared with white people, the adjusted odds of a positive test were 4.75 times greater in black people. ▪ People living in urban areas versus rural areas were 4.59 times more likely to test positive. ▪ People living in more deprived areas were 2.03 times more likely to test positive.
(Drefahl et al., 2020) [40]	Population-based cohort study; Sweden	*N* = 7,775,064 individuals with an average follow-up time of 56 days amounting to a total of 1,189,484 person-years under observation.	Lower education; Lower income; Immigrants.	COVID-19 deaths	▪ In working ages, those in the lowest income tertile were 5.4 times more likely to die from COVID-19 than those in the highest tertile. ▪ In working ages, those with primary or secondary education were more than twice as likely to die relative to those with postsecondary education. ▪ Immigrants from low and middle-income countries were approximately twice as likely to die, as compared to individuals born in Sweden.
(Egede et al., 2020) [41]	Cross-sectional study; US; Milwaukee and Southeast Wisconsin	N = 31,549 adults tested for COVID-19 between March 1 and July 10, 2020 Non-Hispanic White (75.4%) Non-Hispanic Black (19.8%) Hispanic (4.8%)	Non-Hispanic Black; Hispanic.	Confirmed diagnosis; Hospitalization; COVID-19 death.	▪ In adjusted analyses, Blacks were 3.7 times more likely and Hispanics were 3.1 times more likely to have a positive COVID-19 test compared with Whites. ▪ Among those who had a positive COVID-19 test, members of minority groups (Blacks or Hispanics) were two times more likely to be hospitalized compared with Whites after adjustment for demographics and comorbidities. ▪ After adjustment, compared with Whites, Hispanics were two times more likely to die, and there was a small but statistically nonsignificant difference for Blacks.
(Farrell et al., 2020) [42]	Cross-sectional study; Ireland; An Irish hospital	*N* = 257 patients admitted with a diagnosis of SARS-CoV-2 infection diagnosed between March 13 and May 1, 2020. White–Irish *n* = 164 (63.8%) White–other *n* = 44 (17.1%) BAME *n* = 49 (24.1%)	Non-Irish White; Black, Asian or minority ethnic (BAME); Deprivation.	COVID-19 death; ICU admission.	▪ Deprivation was a strong predictor of mortality, even after adjustment for age, one percentage point increase in deprivation was associated with a 5% increase in mortality. ▪ Patients from care homes were 24% more likely to die than community patients after adjusting for age. ▪ Compared with White Irish people, those of other White or BAME ethnicities did not have an increased risk of hospital death, after adjusting for age. This was also the case when ethnicity was broken down into further subcategories. ▪ After adjusting for age, deprivation was not associated with admittance to ICU. ▪ Compared with White Irish patients, all other ethnic groups including other White or other BAME had an approximately fourfold increased risk of ICU admittance after adjusting for age.
(Goyal et al., 2020) [43]	Cross-sectional study; US; An exclusively pediatric drive-through and walk-up SARS-CoV-2 testing site.	*N* = 1000 children (0 and 22 years) tested for SARS-CoV-2 infection non-Hispanic (NH)-white (20.3%) NH-black (30.4%) Hispanic (22.9%) Other, unknown (26.4%)	non-Hispanic Black; Hispanic; Family income;	COVID-19 infection	▪ In comparison with non-Hispanic white children, non-Hispanic Black had 2.3 times higher rates of infection and Hispanic had 6.3 times higher infection. ▪ In comparison with children in the highest median family income quartile, infection rates were 2.6 times higher among children in quartile 3, 2.3 times higher among those in quartile 2, and 2.4 times higher among those in quartile 1.
(Gu et al., 2020) [44]	Retrospective cohort study; US; At the University of Michigan.	*N* = 5698 patients tested or treated for COVID-19 from March 10, 2020, to April 22, 2020. White (65.6%) Black (18.6%) Other, Unknown (15.7%)	Black ethnic group; Populated living area	Hospitalization; ICU admission; COVID-19 death.	▪ Adjusting for age, sex, socioeconomic status, and comorbidity score, Black patients were 72% more likely to be hospitalized compared with White patients. ▪ People living in densely populated areas had 10% higher risk of hospitalization than those non- densely populated areas. ▪ No statistically significant racial differences were found in ICU admission and mortality based on adjusted analysis.
(Hawkins, 2020) [45]	Ecological study; US; Massachusetts	*N* = 87,256 cases of COVID-19 diagnosed in MA between January 1, 2020 and June 10, 2020.	Lower social determinants of health (varia,bles: poverty, Median income, occupations, residents who rented, residents who were uninsured, Unemployment rate)	COVID-19 infection; COVID-19 testing; Confirmed diagnosis.	▪ Cities and towns with a higher percentage of residents living in poverty and lower median incomes tended to have elevated rates of COVID-19. ▪ With respect to employment, cities and towns with more workers employed in the healthcare and social assistance and transportation industries and in service and healthcare support occupations also tended to have higher rates of COVID 19. ▪ Communities with a higher proportion of their population renting and uninsured had elevated COVID-19 rates. ▪ Cities and towns with higher levels of poverty, renting, lack of insurance, lower median incomes, andhigher employment in the transportation industry and service and healthcare support occupations tended to have a higher percentage of positive tests.
(Holmes et al., 2020) [46]	Cross-sectional ecologic design; US; Selected states based on disparity in the previous epidemics.	N = about five states % of population Blacks/AA 13%	Black African American (Black/AA)	Case fatality	▪ Blac/AA had 8% higher risk of death from COVID-19 compared with white population.
(Holtgrave et al., 2020) [47]	Continuum construction; US; New York State.	*N* = 13,990,900 adults 18 years or older of NY Non-Hispanic white (64%) Non-Hispanic Black (16%) Hispanic (19%)	Black non-Hispanic; Hispanic.	COVID-19 death Hospitalization; COVID-19 infection	▪ Compared with white non-Hispanic adults, Black non-Hispanic and Hispanic had approximately doubling risk of infection. ▪ Compared with white non-Hispanic adults, Black non-Hispanics were 4.55 times and Hispanics were 4.36 times at higher risk of hospitalization fro COVID-19. ▪ Compared with white non-Hispanic adults, Black non-Hispanics were 6 times and Hispanics were 4 times at higher risk of death from COVID-19.
(Ioannou et al., 2020) [48]	Longitudinal cohort study; US; Department of Veterans Affairs (VA) national health care system.	*N* = 88,747 patients tested for SARS-CoV-2 between Feburary 28 and May 14, 2020. White (49.6%) Black (41.6%) Other, unknown (8.9%)	Black ethnicity	Hospitalization; Mechanical ventilation; COVID-19 death; Confirmed diagnosis	▪ Compared with individuals who tested negative, those testing positive were more likely to be Black individuals (19,340 [24.6%] vs 4215 [41.6%]). ▪ Compared with White patients, Black patients were 13% more likely to be hospitalized and 52% more likely to receive mechanical ventilation but no more likely to die from COVID-19.
(Joseph et al., 2020) [49]	Single-institution retrospective cohort study; US; Urban quaternary-care academic medical center with affiliated community health centers.	*N* = 326 Patients hospitalized with COVID-19 between March 17, 2020, and April 10, 2020. White (non-Hispanic) n = 116 (35.6%) Black *n* = 27 (8%) Asian n = 10 (3%) Hispanic *n* = 142 (43.6%) Other/unavailable n = 31 (9.5%)	non-White (ie, Hispanic, Black, Asian, or other)	ICU admission; COVID-19 death	▪ Among White or non-Hispanic patients (*n* = 116), 90 (78%) required supplemental oxygen, 36 (31%) were admitted to the intensive care unit, 29 (25%) were intubated, and 27 (23%) died. Among non- White patients (*n* = 210), 153 (73%) required supplemental oxygen, 83 (40%) were admitted to the intensive care unit, 72 (34%) were intubated, and 19 (9%) died.
(Kabarriti et al., 2020) [50]	Cohort study; US; Montefiore Medical Center in New York.	*N* = 9268 patients tested for COVID-19 between March 14 and April 15, 2020. Non-Hispanic White (8.6%) Non-Hispanic Black (32.8%) Hispanic (32.3%) Asian, Other, Unknown (26.3%)	Hispanic and non-Hispanic Black	Confirmed diagnosis; Case fatality rates	▪ Hispanic and non-Hispanic Black patients were approximately 23% and 29%, respectively, more likely to test positive for COVID-19 than White patients. ▪ While controlling for age, sex, socioeconomic status and comorbidities, patients identifying as Hispanic were 23% and non-Hispanic Black were 31% less likely to die compared with non-Hispanic White patients.
(Kaufman et al., 2020) [51]	Cohort study; US; All 50 states and the District of Columbia.	N = 2,331,175 patients with positive SARSCoV- 2 NAAT test result. Black non-Hispanic (12.4%) Hispanic (22.1%) White non-Hispanic (56.1%) Asian, other (9.4%)	Black non-Hispanic community and Hispanic community	Trends of confirmed cases	▪ There was an increasing trend in SARS-CoV-2 NAAT positivity across Black non-Hispanic community progressive quintiles (from 7.8 to 17.2%) and Hispanic community progressive quintiles (from 8.4 to 15.5%) and a decreasing trend across White non-Hispanic community progressive quintiles (from 17.4 to 7.1%).
(Khan et al., 2020) [52]	Prospective cohort study; England, Glasgow, and Lanarkshire; Three acute hospitals.	*N* = 172 hospitalized patients with confirmed COVID-19.	Low Socioeconomic status (SES) based on Scottish index for multiple deprivation (SIMD): more deprived (SIMD 1–5) and less deprived (SIMD 6–10)	Need for intubation; COVID-19 death.	▪ When comparing SIMD 1 to SMID 10, the rate of hospitalization was similar (0.03% vs 0.03%; *P* = .926). ▪ There is no statistically significant difference in both groups for transfer to critical care, intubation, 30-day all-cause mortality, and overall poor outcome.
(Kim et al., 2020) [53]	Cross-sectional study; US; Three hospitals and more than 300 clinics across the Puget Sound.	*N* = 562,242 patients with at least 1 encounter in the system from January 1, 2019, to February 28, 2020. non-English speakers (6.0%)	Non-English speakers	Confirmed diagnosis; Completed testing.	▪ Non-English speakers were overall 16% less likely to have completed testing compared with English-speakers. ▪ Notably, the proportion of positive cases was 4.6-fold higher among non-English speakers overall compared with English speakers.
(Lassale et al., 2020) [54]	Community-based cohort study; England	*N* = 428,494 participants from UK biobank.	Ethnic minority groups	Hospitalization	▪ After adjusting for age and sex, compared to the White population, Black individuals had over a 4-fold increased risk of COVID-19 infection (Hospitalization), and there was a doubling of risk in the Asian group and the ‘other’ non-white group.
(Mahajan & Larkins-Pettigrew, 2020) [55]	Correlation analysis; US; National	*N* = 2886 counties	African–Americans; Asian–Americans	Conformed cases; COVID-19 Death;	▪ A positive correlation existed between percentages of African–Americans living in a county and who have COVID-19, who have died from COVID-19, and case mortality. ▪ There was also a positive correlation between percentage of Asian–Americans living in a county and percentage who have COVID-19 in that county and percentage who have died from COVID-19 in that county. ▪ There was a negative correlation between percentage of Whites living in a county and percentage who have COVID-19 in that county and percentage who have died from COVID-19 in that county.
(Misa et al., 2020) [56]	Retrospective cohort study; US; Northern California Alameda Health System (AHS).	*N* = 526 patients tested for COVID-19. Black (40.7%) Latinx (26.4%) White (15.8%) Asian (7.8%) Other, Unknown (9.4%)	Latinx; Black; Asian	Confirmed diagnosis	▪ Latinx ethnicity had 9.6 times higher likelihood of positive test results compared with non-Latinx patients. In contrast, Black patients had 0.3 times lower risk of positive test results compared with non-Black patients. Asian ethnicity was not associated with odds of confirmed diagnosis.
(Munoz-Price et al., 2020) [57]	Cross-sectional study; US; Froedtert Health and Medical College of Wisconsin (Milwaukee).	*N* = 2595 consecutive adults tested for COVID-19 from March 12 to March 31, 2020. African American (30.2%) White (62.3%) Other (7.4%)	Black; Poverty status (ie, uninsured or receiving Medicaid)	Confirmed diagnosis; Hospitalization; ICU admission; Mechanical Ventilation; COVID-19 death	▪ Regardless of SES, African American patients were 5.37 times more likely to test positive for the virus than persons of other races. ▪ There were no significant associations between poverty status and confirmed diagnosis. ▪ Adjusting for zip code of residence, Black populations were at 1.8 times higher and those with poverty were at 3.8 times higher risk of hospitalization compared with non-Black population and non-poverty status respectively. ICU admission/ ventilation/ mortality ▪ Poor populations were at 3.6 times higher risk of ICU admission. ▪ There were no statistically significant differences between African American patients and patients from other racial groups in ICU admission among those admitted. ▪ Results for mechanical ventilation and death indicated that neither race nor poverty were significantly associated with these outcomes.
(Niedzwiedz et al., 2020) [58]	prospective cohort study; UK; UK Biobank in England.	*N* = 392,116 participants White (94.7) South Asian (1.9) Black (1.6) Others (1.8)	Black and South Asian; Deprivation: 4 quartiles	Confirmed diagnosis	▪ Black and south Asian groups were were 3.35 and 2.42, respectively, times more likely to test positive. ▪ Socioeconomic deprivation was associated with a higher risk of confirmed infection so that the most deprived quartile was 2.19 times at higher risk of confirmed infection compared to least deprived quartile.
(Ojinnaka et al., 2020) [59]	Retrospective study; US; Texas counties	*N* = 254 Texas counties Mean ± SD % non-Hispanic white 55.4 ± 21.04% Black 6.3 ± 6.40% Asian 1.3 ± 2.08% Hispanic 35.3 ± 22.99	African Americans/ Blacks; Hispanics; Unemployment	COVID-19 death (per 100,000)	▪ We observed 5.08 and 4.5% increase in COVID-19 deaths with every 1% increase in the proportion of African Americans/Blacks and Hispanics respectively, but not for Asians. ▪ In addition, there was a 4% increase in COVID-19 deaths/100,000 with every 1% increase in the proportion of unemployed individuals.
(Renelus et al., 2020) [60]	Single-center retrospective cohort study; US; New York City (NYC); university-affiliated NYC hospital.	*N* = 734 patients with COVID-19 Blacks (50.7%), Whites (29.2%), Hispanics (12.5%), Asians (2.7%) Others (4.9)	Black; Hispanics; Asian.	COVID-19 Hospitalization; In-hospital mortality.	▪ Blacks were nearly twice as likely as Whites to require hospitalization for COVID-19. ▪ There was no statistically significant difference in odds for COVID-19 hospitalization between Hispanics and Whites. ▪ After adjusting for age and the other variables, Hispanics along with Asians had doubling hazards of in-hospital mortality. ▪ There was a non-significant increased hazard of in-hospital mortality among Blacks when compared with Whites.
(Rentsch et al., 2020) [61]	Retrospective cohort study; US; Veterans Affairs.	N = 5,834,543 individuals with at least 1 clinical encounter between January 1, 2018, and December 31, 2019. White (73.9%) Black (18.7%) Hispanic (7.5%)	Black; Hispanics.	COVID-19 testing; Confirmed diagnosis; 30-day mortality;	▪ Black individuals were 13% more likely to be tested than Hispanic and 55% higher than whites. ▪ Compared with White populations, Black individuals were 1.9 times more likely and Hispanic individuals were 1.8 times more likely to test positive. ▪ 30-day mortality did not differ by race/ethnicity.
(Rodriguez et al., 2020) [62]	Retrospective data analysis; US; 88 hospitals in the American Heart Association (AHA) COVID-19 CVD Registry.	*N* = 8950 patients hospitalized with COVID-19 from 1/17/2020 to 7/22/2020. non-Hispanic White (59.3%), Black (10.6%), Hispanic (all races) (9%), Asian (4.7%).	Black ethnicity; Hispanics ethnicity; Asian ethnicity.	in-hospital mortality;	▪ Compared with non-Hispanic White patients, risks of death from COVID-19 were 7% less for Black patients and 10% less for Hispanic patients, in contrast, and 31% higher for Asian patients. ▪ Although in-hospital mortality and MACE did not differ by race/ethnicity after adjustment, Black and Hispanic patients bore a greater burden of mortality and morbidity due to their disproportionate representation among COVID-19 hospitalizations.
(Sapey et al., 2020) [63]	Retrospective cohort study; UK; University Hospitals Birmingham NHS Foundation Trust (UHB) in Birmingham.	*N* = 2217 patients with COVID-19 White (69.5) Mixed/multiple (0.8) South Asian/South Asian British (18.5) Black/African/Caribbean/black British (6.0) Other, Unknown (5.2)	Asian; Black.	COVID-19 death while in hospital or post discharge	▪ South Asian ethnic populations were at 30% higher risk of death compared with the rest of the population, after adjusting for age, sex, deprivation and comorbidities, and by propensity score matching. ▪ No significant difference was reported in adjusted model for Black ethnic groups.
(Soares et al., 2020) [64]	cohort of SARSCoV- 2–infected patients; Brazil; Esp’ırito Santo state.	N = 10,713 patients with COVID-19 White (35%) Black/multiracial (42.1%) Asian, indigenous, unknown (22.9%)	Asian ethnic groups; Black/multiracial groups	COVID-19 hospitalization; in-hospital COVID-19 death	▪ Asian ethnic groups were at 1.5 times higher risk of hospitalization compared to Withes. ▪ No significant association were found regarding the risk of hospitalization of Black/multiracial groups. ▪ No significant association was found between race/ethnicity of any group and COVID-19 death in hospitalized patients.
(Yehia et al., 2020) [65]	Cohort study; US; 92 hospitals in 12 states.	N = 11,210 adult hospitalized with COVID-19 between February 19, 2020, and May 31, 2020. White (41%) Black (37.3%) With/other/ missing (21.7%)	Black ethnic groups Insurance	in-hospital COVID-19 death	▪ After adjustment for age, sex, insurance, comorbidities, neighborhood deprivation, and site of care, there was no statistically significant difference in risk of mortality between Black and White patients (hazard ratio, 0.93; 95%CI, 0.80 to 1.09). ▪ Patients with Medicare insurance had 1.5 times, and individuals whose insurance coverage was unknown had 2.2 times higher risk of mortality than those with commercial insurance.
(Zakeri et al., 2020) [66]	Case-control and a cohort study; UK; King’s College Hospital Foundation Trust (KCHFT), which comprises two separate hospitals in south London.	n = consecutive adult patients (age ≥ 18 years) with COVID-19 requiring emergency hospital admission with a primary diagnosis of, between 1 March and 2 June 2020. 872 cases group; *n* = 3488 control group.	Black (African, Caribbean, any other Black); Asian (Indian, Pakistani, Bangladeshi, Chinese, any other Asian),	COVID-19 hospitalization; Inhospital mortality	▪ Adjusting for comorbidities and deprivation, Black ethnicity was associated with 2.2 times and Mixed/Other ethnicity was associated with 2.7 times higher admission risk than white ethnicity. ▪ Asian ethnicity was not associated with higher admission risk than whites. ▪ Black ethnicity was not associated with in-hospital mortality. ▪ Asian ethnicity was associated with 1.71 times higher inhospital mortality but with a large confidence interval (1.15–2.56).
(Lieberman-Cribbin et al., 2020) [67]	Retrospective observational study; US; New York City (NYC)	177 ZIP code Tabulation Areas (ZCTA) in NYC	Hispanic; SES index: household income, gross rent, poverty, education, working class, unemployed, household density.	Confirmed diagnosis; The number of total tests	▪ The number of total tests significantly increased with the increasing proportion of white residents but not with increasing Hispanic composition or SES index score. ▪ The ratio of positive tests to total tests significantly decreased with the increasing proportion of white residents in the ZCTA and with increasing SES index score.
(Loomba et al., 2021) [68]	Retrospective study; US	*N* = 50 states	Black; Asian; Insurance.	COVID-19 death; Testing frequency; COVID-19 infection,.	▪ Lower prevalence of uninsured were associated with greater case frequency on univariate analysis. ▪ Lower prevalence of uninsured were associated with greater testing frequency. ▪ Lower prevalence of uninsured were associated with greater percent mortality on univariate linear regression analyses. ▪ No significant association were found between higher frequency of Black or Asian residents and case frequency, testing, and mortality on univariate analysis.
(Ali et al., 2021) [23]	Retrospective cohort study; Kuwait; Jaber Al-Ahmad Hospital	*N* = 405 patients with COVID-19 between February 24 and May 24, 2020. Arabs (71.6%) South Asians (28.4%)	South Asians group	ICU admission; COVID-19 death.	▪ When compared to Arabs, South Asians also had 6.3 times higher odds of being admitted to the ICU. ▪ South Asian patients showed 7.6 times higher odds of dying from COVID-19.
(Ayoubkhani et al., 2020) [69]	Retrospective cohort study; England and Wales; Residents of England and Wales enumerated in private households.	*N* = 47,872,412 residents White (86.4%) Bangladeshi and Pakistani (3.0%) Black (3.2%) Mixed, other (7.5%)	Black; Bangladeshi/Pakistani, Indian, Mixed and Other ethnic backgrounds	COVID-19 death (age-standardized mortality rates (ASMRs))	▪ The ASMRs of COVID-19 mortality were greatest among individuals identifying as Black and lowest among those identifying as White. ▪ The rate of COVID-19 death was 3.13 times greater for Black males than for White males, and 2.40 times greater for Black females than White females. ▪ People of Bangladeshi/Pakistani, Indian, Mixed and Other ethnic backgrounds also had raised rates of death involving COVID-19 compared with those of White ethnicity.
(Baena-Diez et al., 2020) [70]	Ecological study; Spain; Barcelona.	N = 10 districts of the city of Barcelona	Poverty (lower income districts)	COVID-19 incidence (per 10,000)	▪ Districts with the lowest mean income had the highest incidence of COVID-19 per 10,000 inhabitants; in contrast, those with the highest income had the lowest incidence. ▪ The district with the lowest income had 2.5 times greater incidence of the disease, compared with the highest-income district.
(Boserup et al., 2020) [71]	cross-sectional study; US; 48 states/ regions	*N* = 173 counties spanning 37 states from March 1, 2020, to July 11, 2020.	Deprivation; Unemployment; English proficiency.	COVID-19 death (per 100,000)	▪ The predicted number of white COVID-19 deaths was 4% higher in counties with an increased percentage of households without a vehicle. ▪ The predicted number of black COVID-19 deaths was 3% higher in counties with an increased percentage of households without a vehicle. Conversely, it was 16% lower in counties with higher unemployment rates. ▪ The predicted number of Hispanic COVID-19 deaths/ 100,000 population was 22% higher in counties with an increased percentage of persons (age ≥ 5 years) who speak English “less than well” and 3% higher in counties with an increased percentage of households with no vehicle available, conversely, was 14% lower in counties with higher unemployment rates.
(DiMaggio et al., 2020) [72]	Ecological study; US; New York	*N* = 177 ZIP code Tabulation Area (ZCTAs) Proportion black 0.23 Proportion Hispanic 0.12 (0.05)	Black/African American residents; Household income; Housing density; Non-English proficiency.	Confirmed diagnosis	▪ There was a nearly five-fold increase in the risk of a positive COVID-19 test associated with the proportion of black/African American residents. ▪ For each unit increase in a standardized measure of median household income in a ZCTA, there was an approximately 46% decrease in the number of positive COVID-19 tests. ▪ Increases in the housing density was associated with an approximate doubling of risk. ▪ Proportion of persons not speaking English, and the proportion of persons on public assistance were not associated with positive COVID-19 testing rates.
(Fielding-Miller et al., 2020) [73]	Observational study; US; All 50 states	*N* = 3024 counties from all 50 states	Poverty; Uninsured; Non-English; Language; Farm worker; Occupation.	COVID-19 death (per 100,000)	▪ The percentage of non-English speaking households in a county was significantly associated with higher rates of death across all counties. ▪ The percentage of uninsured individuals was associated with fewer reported COVID-19 deaths across all counties. ▪ Poverty was associated with fewer reported deaths across all Mid-Atlantic counties and in non-urban Mid-Atlantic counties, but with more reported deaths in all counties in the East South Central region and in non-urban counties in the same region.
(Figueiredo et al., 2020) [24]	Ecological study; Brazil	N = all Brazilian Federative Units (FU)	Household income; Overcrowded households;	Incidence; COVID-19 death.	▪ Adjusting to lethality and Gini Index of household income, one unit increase in household’s size results in 0.35% increase in risk of COVID-19 infection. ▪ Adjusting for lethality and overcrowded households, one unit increase in the Gini Index of household income results in 0.36% increase in risk of COVID-19 infection. ▪ Adjusting for lethality and Gini Index of household income, one unit increase in household’s size results in 0.35% increase in risk of COVID-19 death. ▪ Adjusting for lethality and overcrowded households, one unit increase in the Gini Index of household income results in 0.41% increase in risk of COVID-19 death.
(Hawkins et al., 2020) [74]	Cohort study; US; All 50 states.	*N* = 3127 counties	Black residents; Education level; Unemployment; Poverty.	Confirmed cases (100,000 population); Fatality (per 100,000)	▪ The median percentage of black Americans was 4 times higher in severely distressed counties compared with less distressed counties. ▪ The median percentage of uninsured individuals was 49% higher in severely distressed counties compared with less distressed counties. ▪ A higher number of cases were associated with lower education level, higher proportion of black Americans, higher income and lower poverty rate. ▪ Higher COVID-19 mortality was associated with higher income but lower education, higher employment rate, and higher proportion of black Americans.
(Hu et al., 2020) [75]	Observational study; US; city/town level in Massachusetts	*N* = 6,547,785 people of Massachusetts Non-Hispanic Black (2.75%) Hispanic (4.83%) Asian (3.60%) Non-Hispanic White (91.06%)	Hispanic and Non-Hispanic Black/African Americans; Poverty; Overcrowding households,	Incidence; Testing site access	▪ With a parameter estimate of −66.217 (SEM), the rate of the population below the poverty level had a significantly negative influence on the COVID-19 incidence rate. ▪ Income inequality had a nonsignificant and negative impact on the COVID-19 incidence rate. ▪ One-point increase in the rate of households with more than 1 occupant per room was associated with a 157.385-point increase in COVID-19 incidence rate. ▪ Non-Hispanic Black had the lowest weighted travel time of 5.69 min to the testing sites, followed by the Hispanic, Asian, and White groups. ▪ Higher Hispanic and Black/African American segregations are more likely to be associated with a higher COVID-19 incidence rate.
(Madhav et al., 2020) [76]	Observational study; US; Neighborhoods in Louisiana.	*N* = 4138 population (64 parishes (counties) and 1148 census)	Deprivation (based on Area Deprivation Index (ADI) Quintiles (Q)); Urban.	Rate of COVID-19 infection	▪ The most deprived neighborhoods (5th quintile) had a 30% higher rate of COVID-19 infection compared to those in the least deprived (1st quintile) neighborhoods. ▪ Adjusting for the effect of urban residence, those living in the most deprived neighborhood (5th quintile) had a 39% higher rate of COVID-19 infection compared to those living in the least deprived neighborhood (1st quintile). ▪ Urban location was also significantly associated with COVID-19 infection with 32% higher rate of COVID-19 infection compared to non-urban area.
(Raine et al., 2020) [77]	Cross-sectional study; US; National.	US population; 45 out of the 50 US states White (61.10%) Latinx (17.80%) Black (12.30%) Asian (5.40%) American Indian or Alaskan Native (AIAN) (0.70%) Native Hawaiian or Pacific Islander (NHPI) (0.2%) Other/Unknown (2.6%)	Hispanic/Latinx, American Indian/Alaskan Native, Native Hawaiian/Pacific Islanders, and Black people	Incidence; COVID-19 death.	▪ On a national level, Hispanic/Latinx, American Indian/Alaskan Native, Native Hawaiian/Pacific Islanders, and Black people had Representation Quotients (RQs) > 1, indicating that these groups are over-represented in COVID-19 incidence. ▪ Dramatic racial and ethnic variances in state-level incidence and mortality RQs were also observed.
(Ossimetha et al., 2021) [78]	Observational study; US; Counties with at least one COVID-19 case	*N* = 2664 counties	Lower social deprivation index (SDI)	COVID-19 infection (per 1000); COVID-19 death	▪ Medium- and high-SDI counties had 1.39 and 2.56 more SARS-CoV-2 cases/1000 population compared with low-SDI counties, respectively. ▪ Deaths per capita were also significantly higher for higher-SDI counties.
(Khanijahani & Tomassoni, 2021) [80]	Retrospective observational study; US; All 50 states and the District of Columbia	*N* = 3142 county Mean (SD) % Black-concentrated 13.3 (27.2)	Black population (Black-concentrated if 25% or more were Black); Disadvantaged area	COVID-19 deaths (per 100,000)	▪ For every 10% increase in the percentage of county population residing in concentrated disadvantage and Black-concentrated tracts, the rate for confirmed COVID-19 deaths per 100,000 population increases by a factor of 1.14.
(Khanijahani, 2021) [79]	Retrospective observational study; US; 3142 counties in 50 states and the District of Columbia	N = 3142 counties in 50 states and the District of Columbia Mean (SD) % Hispanic 9.3 (13.8) % Black 9.9 (14.7)	Black race; Hispanic ethnic; Uninsured residents; Household size; Household income; Education	COVID-19 cases; COVID-19 deaths	▪ 1% increase in proportion of the Hispanic or Black population increase the confirmed cases by 0.68 and 0.69% respectively. ▪ 1% increase in proportion of the Hispanic or Black population increase the COVID-19 death by 0.65 and 1.57%. ▪ Higher proportions of adults with no high school diploma was associated with higher COVID-19 cases (R = 0.32) and deaths (R = 0.37). ▪ One unit higher median household income was associated with 0.89% higher deaths per population unit. ▪ There were no significant association between the average household size and % Uninsured population and confirmed COVID-19 deaths and confirmed COVID-19 cases.
(Weech-Maldonado et al., 2021) [26]	Cross-sectional study; US; All US nursing homes.	*N* = 12,914 nursing home	Minority (not specified): categorizing as high-minority nursing homes and nursing facilities with no minorities.	COVID-1 death	▪ After controlling for interstate differences, facility-level resident characteristics, resource availability, and organizational characteristics, high-minority nursing homes had 61% more COVID-19 deaths as compared to nursing facilities with no minorities.

A few studies examined the access to testing for COVID-19 among different racial/ethnic groups. One study showed that Black populations were more likely to get tested for COVID-19 than Hispanic and White populations [61]. One study did not report significant differences between race/ethnicity and testing for COVID-19 [68]. One study reported that racial/ethnic minority groups, including non-Hispanic Black, Hispanic, and Asian populations had lower population-weighted travel time to the testing sites than White populations [75]. In contrast, another study showed that areas with a higher proportion of the White population had a higher number of total tests [67]. One study showed that compared to other racial/ethnic groups (including Whites, Asians, and Hispanics), a smaller percentage of African Americans were tested for COVID-19 in an ambulatory setting. Most African Americans were tested in hospitals, either in the emergency department or during inpatient hospitalization [34].

Nine studies [33, 34, 41, 44, 47, 48, 54, 57, 66] showed that the racial/ethnic minority populations were more likely to be hospitalized for COVID-19 than majority racial/ethnic groups or the rest of the population. Nonetheless, one study from the US reported inconsistent results showing that while the Blacks were about twice as likely as Whites to be hospitalized for COVID-19, no statistically significant differences were found comparing Hispanics and non-Hispanic Whites [60]. Another study from the US found that although Asians were more likely to require hospitalization for COVID-19 than Whites, no statistically significant differences were found between Blacks or multiracial groups and Whites [64].

Three studies showed that racial/ethnic minority populations had a disproportionately higher ICU admissions rate than the rest of the populations [23, 42, 49]. One study from Ireland, for instance, reported that compared with White Irish patients, all other ethnic groups had an approximately fourfold increased risk of ICU admittance after adjusting for age [42]. Another study from Kuwait showed that South Asians had approximately six times higher odds of being admitted to the ICU when compared to Arabs [23]. One study from the US also reported that a smaller percent of White or non-Hispanic patients were admitted to ICU compared with non-White patients [49]. In contrast, two studies did not report a statistically significant association between race/ethnicity differences and risk of ICU admission for COVID-19 [44, 57]. Moreover, whereas one study from the US showed that Black patients were more likely than Whites to receive mechanical ventilation [48], another study from the US showed that there were no significant racial/ethnic differences in receiving mechanical ventilation [57]. A summary of the results is presented in Table 2.

### Socioeconomic disparities and COVID-19 outcomes

Overall, 28 studies examined the association between COVID-19 outcomes and SES characteristics. In this regard, studies showed that poor housing conditions [38], living in poverty [24, 45, 75] or deprivation [76, 78], employment in the healthcare and social assistance and transportation industries [45], lack of insurance [45, 74], household overcrowding [24, 75], lower household income [24, 37, 43, 45, 70], no or lower level of education [74, 79], and urban residency [76] were associated with a higher risk of COVID-19 incidence/infection. Some studies showed contradicting results, reporting that areas with higher median income [37, 74] or lower poverty [74] had a higher infection rate. One study from the US, on the other hand, showed that areas with a higher proportion of uninsured individuals were associated with a lower rate of infection [68]. Moreover, a few studies demonstrated that households’ income differences [75], average household size [79], and lack of insurance [37, 79] were not significantly associated with the risk of COVID-19 incidence.

Regarding COVID-19 deaths, studies showed that a low level of education [40, 74, 79], poverty [24, 37], poor housing conditions [38], low family income [40], deprivation [42, 71, 78, 79], speaking in a language other than the national language in a country [71, 73], household overcrowding [24], being an immigrant [40], and unemployment [59, 71] were associated with a higher risk of death from COVID-19. Nevertheless, there were some contradicting results as well. For example, higher risks of death from COVID-19 was associated with lower rates of unemployment [74], a lower proportion of uninsured individuals in a region [68, 73], and higher household income [74, 79]. A few studies reported that low SES [52], poverty [57], large household size [79], a higher proportion of uninsured individuals in a region [79], and deprivation [52] were not associated with higher risks of COVID-19 death. However, one study reported that living in poverty was associated with fewer reported deaths in some counties and more reported deaths in some other counties [73].

Several studies corroborate that people living in urban areas [27], areas with higher levels of poverty [45], deprivation [27, 58], rental housing [45], lack of insurance [45], lower household income [45, 72], higher employment in the transportation and healthcare industries [45], and overcrowded households [53, 72] were more likely to have positive COVID-19 test results. However, there was some inconsistent evidence as well. For example, one study from the US did not report a statistically significant association between poverty status and confirmed diagnosis [57]. Another study from the US reported that higher median household income was associated with a higher likelihood of positive COVID-19 tests [74].

One study showed that the likelihood of getting a COVID-19 test was lower for non-English speakers than English speakers [53]. In one study, the number of total tests was not associated with the SES index, which was a composite score made of SES variables including household income, gross rent, poverty, education, working class, unemployment, and household density [67]. Another study showed that testing frequency was higher in areas with fewer uninsured individuals [68]. Three studies from the US showed that people living in densely populated areas [44] or areas with a higher poverty level [57] were at a higher risk of hospitalization for COVID-19. Two studies showed that people with Medicaid/Medicare or no reported insurance had a higher risk of hospitalization [34] and mortality [65] than those with commercial insurance. One study did not find an association between living in deprivation and hospitalization [52].

Regarding ICU admission, two studies did not report an association with deprivation [42, 52], and one study did not show an association with the SES index [52]. However, one study showed that individuals living in higher poverty levels were at higher risk of ICU admission for COVID-19 [57]. Regarding mechanical ventilation, one study did not report a statistically significant association between the need for mechanical ventilation and living in poverty among patients with COVID-19 [57].

## Discussion

This systematic review studied the racial/ethnic and SES disparities in health outcomes and access to health resources during the COVID-19 pandemic. Due to the inconsistencies among studies, especially regarding different definitions for racial/ethnic minority groups and low-SES, it was challenging to summarize the results. Generally, evidence showed that racial/ethnic minority populations were at a higher risk of infection, having positive test results, and hospital admissions for COVID-19 [32, 33, 37, 43, 47]. Studies on the risk of death from COVID-19 among different racial/ethnic populations and those with different SES showed different results. Regarding access to testing, the inconsistency was much more evident. Two studies showed greater access to testing for racial/ethnic minority populations [61, 75], two studies yielded reverse results [34, 67], and one study showed no significant differences [68]. We also found similar results for ICU admission following the COVID-19 infection.

Nineteen out of 28 studies showed that people from low SES, including those with poor housing conditions, poverty, household overcrowding, and lower level of education, were at higher risk of infection [24, 38, 43, 45, 68, 70, 75, 76, 78, 80], death [24, 37, 40, 42, 71, 74, 78–80] and confirmed diagnosis [27, 45, 58, 81]. However, this was not always the case [37, 52, 57, 73–75, 80, 82]. For instance, according to the results of four studies, areas with a higher median income had a higher rate of infection, death, or confirmed diagnosis [37, 73, 74, 80].

Lack of insurance and unemployment affected the outcomes in different ways. Out of 28 studies, four studies reported a positive association between lack of insurance and COVID-19 incidence [45, 74] and death [68, 73]. Moreover, two studies did not report a notable association between lack of insurance and risk of COVID-19 death and COVID-19 incidence/infection [37, 79]. Just one study reported lack of insurance as a predictor of decreased risk of COVID-19 incidence [68]. Out of 28 studies on SES, two studies showed that a higher rate of unemployment was associated with a higher risk of death from COVID-19 [59, 71]. In contrast, one study reported that a higher risk of death from COVID-19 was associated with lower rates of unemployment [74]. One possible explanation for the inconsistent results regarding unemployment and lack of insurance is that information regarding the health outcomes of unemployed and uninsured individuals might not be wholly documented due to the underutilization of services. More studies are needed to explore the impacts of unemployment and lack of insurance on patients with COVID-19.

Though racial/ethnic minority groups were frequently identified as the most vulnerable populations during the epidemics, they are exceptionally vulnerable in the COVID-19 pandemic because the transmission of the infection is strongly associated with the background and socioeconomic characteristics of individuals. There are some potential reasons for the higher incidence and severity of COVID-19 in racial/ethnic minority groups and individuals from low SES. When speaking about SES, we generally focus on people’s occupation, income, and education level [83]. The risk of the transmission of COVID-19 in professions with constant in-person interactions is higher than in other professions. As a result, the incidence of the disease among service industry workers is higher [84]. Additionally, people with low SES are more likely to experience work stress, which increases the risk of cardiovascular disease [85] and disrupts the function of the immune system [86], consequently resulting in lower resistance to COVID-19. Low household income can influence the housing conditions of the individuals to increase the risk of the spread of infectious diseases among those living in small and overcrowded housing units [6]. Lower education levels may increase the COVID-19 severity indirectly by behavioral pathways, poor diet, smoking and other risk factors, and problems with effectively navigating healthcare systems [87]. Racial/ethnic minorities are usually impacted by higher poverty rates, are economically disadvantaged, and are more likely to work in jobs unsuitable for remote working [58, 88–90].

This systematic review can inform policymakers, practitioners, and researchers of the potential inequalities in health outcomes and access to services, helping them adopt effective strategies to manage COVID-19 as a public health emergency. There is no doubt that most of the racial/ethnic minorities and those from low SES are more vulnerable to COVID-19; therefore, the information provided by this review study can provide authorities with insight into the inequalities that COVID-19 poses to these vulnerable populations.

### Limitations

We acknowledge the limitations of this study. First, race/ethnicity and SES data might have been incomplete (or inaccurate in some cases) in medical records and datasets, and their accuracy evaluation were not reported [6]. This could have made it difficult for researchers to measure health disparities in COVID-19 and have resulted in major inconsistencies among the studies, making it challenging to congregate the results. Additionally, many LMICs do not have appropriate information systems to provide quality data [25], especially regarding case ascertainment and SES information, and in some cases, the assignment of cause-of-death. As a result, there are fewer studies from LMICs. Second, the diagnostic tests and degree of accuracy (sensitivity and specificity) might vary from study to study, resulting in the misclassification of infected and healthy individuals. Finally, we only included the English-language studies in the review. As a result, we might have missed several studies from non-English language publications. Heterogenous populations and different levels of study design (individual patient-level or spatial analysis) can also contribute to contradicting findings.

In order to mitigate the limitation to some degree, we extracted detailed information from each study and summarized them in the data extraction sheet. This would help readers to review the summary results of each study in line with their potential limitations. Despite the aforementioned limitations, we believe that this systematic review can provide insight into the status of the disparity in the COVID-19 and can contribute to future research in the field.

## Conclusion

Although we observed the inconsistencies among the included studies, most of these studies showed that racial/ethnic minority populations in a region are at greater risk of COVID-19 infection, hospitalization, confirmed diagnosis, and mortality. Additionally, most of the studies cited factors such as low level of education, poverty, poor housing conditions, low family income, speaking in a language other than the national language in a country, and household overcrowding as risk factors of COVID-19 incidence, death, and confirmed diagnosis. The potential impact of lack of insurance and unemployment on the outcome measures such as the need for mechanical ventilation, ICU admission, and access to testing for COVID-19 were limited and inconsistent. Further studies are needed to fill the gaps. This systematic review also revealed a major incongruity in the definition of minority ethnic/race groups and SES among the studies.

## Supplementary Information

**Additional file 1:.** Search strategies.

**Additional file 2:.** Data extraction table.

**Additional file 3:.** Assessment of methodological quality.

## Data Availability

The datasets used and/or analyzed during the current study are available from the corresponding author on reasonable request.

## Abbreviations

COVID-19: Coronavirus Disease 2019
WHO: World Health Organization
SES: Socioeconomic status
LMICs: Low- and Middle-Income Countries
PRISMA: Preferred Reporting Items for Systematic Reviews and Meta-Analyses
JBI: Joanna Briggs Institute
US: United States
ICU: Intensive Care Unit
