# Supplementary material for: A systematic review of racial/ethnic and socioeconomic disparities in COVID-19
Source: Int J Equity Health. 2021 Nov 24;20:248. doi: 10.1186/s12939-021-01582-4 (PMC8611382; doi:10.1186/s12939-021-01582-4)
Supplement: Supplementary file 1 — Additional file 1:. Search strategies. [file 12939_2021_1582_MOESM1_ESM.pdf]

Additional file 1. Search strategies (November 20, 2020)

Pubmed

N		Queries	Results
#1		Search: ((((((((("2019 novel coronavirus"[Title/Abstract]) OR ("COVID19"[Title/Abstract])) OR ("COVID-19"[Title/Abstract])) OR ("2019-novel CoV"[Title/Abstract])) OR ("SARS-cov-2"[Title/Abstract])) OR ("SARS-CoV2"[Title/Abstract])) OR ("SARSCoV2"[Title/Abstract])) OR ("SARSCoV-2"[Title/Abstract])) OR ("severe acute respiratory syndrome coronavirus 2"[Title/Abstract])) OR ("2019-ncov"[Title/Abstract])) OR ("coronavirus disease 2019"[Title/Abstract])	72,027
#2		Search: ("severe acute respiratory syndrome coronavirus 2"[Supplementary Concept]) OR ("COVID-19"[Supplementary Concept])	36,869
#3	#1 OR #2	Search: (("severe acute respiratory syndrome coronavirus 2"[Supplementary Concept]) OR ("COVID-19"[Supplementary Concept])) OR (((((((((("2019 novel coronavirus"[Title/Abstract]) OR ("COVID19"[Title/Abstract])) OR ("COVID-19"[Title/Abstract])) OR ("2019-novel CoV"[Title/Abstract])) OR ("SARS-cov-2"[Title/Abstract])) OR ("SARS-CoV2"[Title/Abstract])) OR ("SARSCoV2"[Title/Abstract])) OR ("SARSCoV-2"[Title/Abstract])) OR ("severe acute respiratory syndrome coronavirus 2"[Title/Abstract])) OR ("2019-ncov"[Title/Abstract])) OR ("coronavirus disease 2019"[Title/Abstract]))	73,805
#4		Search: (((((((((((((((((((((((ethnic*[Title/Abstract]) OR (disparit*[Title/Abstract])) OR ("health status disparities"[MeSH Terms])) OR ("healthcare disparities"[MeSH Terms])) OR (race*[Title/Abstract])) OR (minorit*[Title/Abstract])) OR ("minority health"[MeSH Terms])) OR ("population characteristics"[MeSH Terms])) OR ("ethnic groups"[MeSH Terms])) OR ("race factors"[MeSH Terms])) OR (inequit*[Title/Abstract])) OR (Socioeconomic*[Title/Abstract])) OR ("socioeconomic factors"[MeSH Terms])) OR ("health equity"[MeSH Terms])) OR ("poverty"[MeSH Terms])) OR ("poverty areas"[MeSH Terms])) OR ("racism"[MeSH Terms])) OR (poverty[Title/Abstract])) OR (inequalit*[Title/Abstract])) OR ("social determinants of health"[MeSH Terms])) OR (indigence*[Title/Abstract])) OR (discrimination[Title/Abstract])) OR (equit*[Title/Abstract])) OR (income[Title/Abstract])) OR ("social class"[Title/Abstract])) OR ("social class"[MeSH Terms])	2,507,121
#5		Search: ((((((((((((((((((((access*[Title/Abstract]) OR ("health services accessibility"[MeSH Terms])) OR (hospitalization*[Title/Abstract])) OR ("hospitalization"[MeSH Terms])) OR (mortality*[Title/Abstract])) OR ("mortality"[MeSH Terms])) OR (morbidity*[Title/Abstract])) OR ("morbidity"[MeSH Terms])) OR ("diagnostic tests"[Title/Abstract])) OR ("covid-19 diagnostic testing"[Supplementary Concept])) OR ("new case*"[Title/Abstract])) OR ("basic reproduction number"[Title/Abstract])) OR ("basic reproduction number"[MeSH Terms])) OR ("intensive care"[Title/Abstract])) OR ("prevalence"[Title/Abstract])) OR ("prevalence"[MeSH Terms])) OR ("incidence"[MeSH Terms])) OR (incidence[Title/Abstract])) OR ("case fatality rate"[Title/Abstract])) OR (test*[Title/Abstract])) OR (diagnos*[Title/Abstract])) OR ("death rate"[Title/Abstract])) OR (death rate[Title/Abstract])	7,803,168
#6	#3 AND #4 AND #5		5,588
		English	5,458

CINAHL

N		Queries	Results
S1		TI "2019 novel coronavirus" OR TI "COVID19" OR TI "COVID-19" OR TI "COVID 2019" OR TI "2019-novel CoV" OR TI "SARS-cov-2" OR TI "SARS-CoV2" OR TI "SARSCoV2" OR TI "severe acute respiratory syndrome coronavirus 2" OR TI "coronavirus disease 2019" OR TI "2019ncov" OR MW "COVID-19"	(25,351)

S2		AB "2019 novel coronavirus" OR AB "COVID19" OR AB "COVID-19" OR AB "coronavirus disease-19" OR AB "2019-novel CoV" OR AB "SARS-cov-2" OR AB "SARS-CoV2" OR AB "SARSCoV2" OR AB "severe acute respiratory syndrome coronavirus 2" OR AB "coronavirus disease 2019" OR AB "2019ncov"	(14,068)
S3	S1 OR S2		(27,177)
s4		TI ethnic* OR TI disparit* OR TI race* OR TI minorit* OR TI inequit* OR TI socioeconomic* OR AB ethnic* OR AB disparit* OR AB race* OR AB minorit* OR AB inequit* OR AB socioeconomic*	(165,263)
S5		TI poverty OR TI inequalit* OR TI "social determinants of health" OR TI indigence* OR TI discrimination OR TI equit* OR AB poverty OR AB inequalit* OR AB "social determinants of health" OR AB indigence* OR AB discrimination OR AB equit*	(65,290)
S6		TI income OR TI "social class" OR AB income OR AB "social class" OR MW "socioeconomic" OR MW "social class" OR MW "disparities" OR MW "healthcare disparities" OR MW "health status disparities" OR MW "ethnic groups" OR MW "race factors"	(197,266)
S7	S4 OR S5 OR S6		(333,713)
S8		TI access* OR TI hospitalization* OR TI mortality* OR TI morbidity* OR TI "diagnostic tests" OR TI "new case*" OR AB access* OR AB "diagnostic tests" OR AB hospitalization* OR AB mortality* OR AB morbidity* OR AB "new case*"	(431,673)
S9		TI "basic reproduction number" OR TI "intensive care" OR TI "prevalence" OR TI "incidence" OR TI "case fatality rate" OR TI test* OR AB "basic reproduction number" OR AB "intensive care" OR AB "prevalence" OR AB "incidence" OR AB "case fatality rate" OR AB test*	(927,225)
S10		TI diagnos* OR TI "death rate" OR AB diagnos* OR AB "death rate" OR MW "health services accessibility" OR MW "health care delivery" OR MW "diagnostic tests" OR MW "intensive care units" OR MW "mortality" OR MW "morbidity" OR MW "hospitalization" OR MW "basic reproduction number"	(909,507)
S11	S8 OR S9 OR S10		(1,786,367)
S12	S3 AND S7 AND S11		(839)
S13		Limiters - Published Date: 20191201-; English Language<br>Expanders - Apply equivalent subjects<br>Search modes - Boolean/Phrase	(836)

SCOPUS

**N**		**Queries**	**Results**
1		(TITLE-ABS-KEY (*"2019 novel coronavirus"*) OR TITLE-ABS-KEY (*"COVID19"*) OR TITLE-ABS-KEY (*"COVID-19"*) OR TITLE-ABS-KEY (*"COVID 2019"*) OR TITLE-ABS-KEY (*"2019-novel CoV"*) OR TITLE-ABS-KEY (*"SARS-cov-2"*) OR TITLE-ABS-KEY (*"SARS-CoV2"*) OR TITLE-ABS-KEY (*"SARSCoV2"*) OR TITLE-ABS-KEY (*"SARSCoV-2"*) OR TITLE-ABS-KEY (*"severe acute respiratory syndrome coronavirus 2"*) OR TITLE-ABS-KEY (*"2019-ncov"*) OR TITLE-ABS-KEY (*"coronavirus disease 2019"*) OR TITLE-ABS-KEY (*"coronavirus disease-19"*) OR TITLE-ABS-KEY (*"2019ncov"*) OR TITLE-ABS-KEY (*"SARS coronavirus 2"*))	79,751
2		(TITLE-ABS-KEY (*ethnic**) OR TITLE-ABS-KEY (*disparit**) OR TITLE-ABS-KEY (*race**) OR TITLE-ABS-KEY (*minorit**) OR TITLE-ABS-KEY (*inequit**) OR TITLE-ABS-KEY (*socioeconomic**) OR TITLE-ABS-KEY (*poverty*) OR TITLE-ABS-KEY (*inequalit**) OR TITLE-ABS-KEY (*"social determinants of health"*) OR TITLE-ABS-KEY (*indigence**) OR TITLE-ABS-KEY (*discrimination*) OR TITLE-ABS-KEY (*equit**) OR TITLE-ABS-KEY (*income*) OR TITLE-ABS-KEY (*"social class"*))	2,162,703
3		(TITLE-ABS-KEY (*access**) OR TITLE-ABS-KEY (*hospitalization**) OR TITLE-ABS-KEY (*mortality**) OR TITLE-ABS-KEY (*morbidity**) OR TITLE-ABS-KEY (*"diagnostic tests"*) OR TITLE-ABS-KEY (*"new case*"*) OR TITLE-ABS-KEY (*"basic reproduction*	18,418,225

		number") OR TITLE-ABS-KEY (*"intensive care"*) OR TITLE-ABS-KEY (*"prevalence"*) OR TITLE-ABS-KEY (*"incidence"*) OR TITLE-ABS-KEY (*"case fatality rate"*) OR TITLE-ABS-KEY (*test**) OR TITLE-ABS-KEY (*diagnos**) OR TITLE-ABS-KEY (*"death rate"*) OR TITLE-ABS-KEY (*complication**))	
4	#1 AND #2 AND 3	((TITLE-ABS-KEY (*"2019 novel coronavirus"*) OR TITLE-ABS-KEY (*"COVID19"*) OR TITLE-ABS-KEY (*"COVID-19"*) OR TITLE-ABS-KEY (*"COVID 2019"*) OR TITLE-ABS-KEY (*"2019-novel CoV"*) OR TITLE-ABS-KEY (*"SARS-cov-2"*) OR TITLE-ABS-KEY (*"SARS-CoV2"*) OR TITLE-ABS-KEY (*"SARSCoV2"*) OR TITLE-ABS-KEY (*"SARSCoV-2"*) OR TITLE-ABS-KEY (*"severe acute respiratory syndrome coronavirus 2"*) OR TITLE-ABS-KEY (*"2019-ncov"*) OR TITLE-ABS-KEY (*"coronavirus disease 2019"*) OR TITLE-ABS-KEY (*"coronavirus disease-19"*) OR TITLE-ABS-KEY (*"2019ncov"*) OR TITLE-ABS-KEY (*"SARS coronavirus 2"*))) AND ((TITLE-ABS-KEY (*ethnic**) OR TITLE-ABS-KEY (*disparit**) OR TITLE-ABS-KEY (*race**) OR TITLE-ABS-KEY (*minorit**) OR TITLE-ABS-KEY (*inequit**) OR TITLE-ABS-KEY (*socioeconomic**) OR TITLE-ABS-KEY (*poverty*) OR TITLE-ABS-KEY (*inequalit**) OR TITLE-ABS-KEY (*"social determinants of health"*) OR TITLE-ABS-KEY (*indigence**) OR TITLE-ABS-KEY (*discrimination*) OR TITLE-ABS-KEY (*equit**) OR TITLE-ABS-KEY (*income*) OR TITLE-ABS-KEY (*"social class"*))) AND ((TITLE-ABS-KEY (*access**) OR TITLE-ABS-KEY (*hospitalization**) OR TITLE-ABS-KEY (*mortality**) OR TITLE-ABS-KEY (*morbidity**) OR TITLE-ABS-KEY (*"diagnostic tests"*) OR TITLE-ABS-KEY (*"new case*"*) OR TITLE-ABS-KEY (*"basic reproduction number"*) OR TITLE-ABS-KEY (*"intensive care"*) OR TITLE-ABS-KEY (*"prevalence"*) OR TITLE-ABS-KEY (*"incidence"*) OR TITLE-ABS-KEY (*"case fatality rate"*) OR TITLE-ABS-KEY (*test**) OR TITLE-ABS-KEY (*diagnos**) OR TITLE-ABS-KEY (*"death rate"*) OR TITLE-ABS-KEY (*complication**)))	3,116
5		((TITLE-ABS-KEY (*"2019 novel coronavirus"*) OR TITLE-ABS-KEY (*"COVID19"*) OR TITLE-ABS-KEY (*"COVID-19"*) OR TITLE-ABS-KEY (*"COVID 2019"*) OR TITLE-ABS-KEY (*"2019-novel CoV"*) OR TITLE-ABS-KEY (*"SARS-cov-2"*) OR TITLE-ABS-KEY (*"SARS-CoV2"*) OR TITLE-ABS-KEY (*"SARSCoV2"*) OR TITLE-ABS-KEY (*"SARSCoV-2"*) OR TITLE-ABS-KEY (*"severe acute respiratory syndrome coronavirus 2"*) OR TITLE-ABS-KEY (*"2019-ncov"*) OR TITLE-ABS-KEY (*"coronavirus disease 2019"*) OR TITLE-ABS-KEY (*"coronavirus disease-19"*) OR TITLE-ABS-KEY (*"2019ncov"*) OR TITLE-ABS-KEY (*"SARS coronavirus 2"*))) AND ((TITLE-ABS-KEY (*ethnic**) OR TITLE-ABS-KEY (*disparit**) OR TITLE-ABS-KEY (*race**) OR TITLE-ABS-KEY (*minorit**) OR TITLE-ABS-KEY (*inequit**) OR TITLE-ABS-KEY (*socioeconomic**) OR TITLE-ABS-KEY (*poverty*) OR TITLE-ABS-KEY (*inequalit**) OR TITLE-ABS-KEY (*"social determinants of health"*) OR TITLE-ABS-KEY (*indigence**) OR TITLE-ABS-KEY (*discrimination*) OR TITLE-ABS-KEY (*equit**) OR TITLE-ABS-KEY (*income*) OR TITLE-ABS-KEY (*"social class"*))) AND ((TITLE-ABS-KEY (*access**) OR TITLE-ABS-KEY (*hospitalization**) OR TITLE-ABS-KEY (*mortality**) OR TITLE-ABS-KEY (*morbidity**) OR TITLE-ABS-KEY (*"diagnostic tests"*) OR TITLE-ABS-KEY (*"new case*"*) OR TITLE-ABS-KEY (*"basic reproduction number"*) OR TITLE-ABS-KEY (*"intensive care"*) OR TITLE-ABS-KEY (*"prevalence"*) OR TITLE-ABS-KEY (*"incidence"*) OR TITLE-ABS-KEY (*"case fatality rate"*) OR TITLE-ABS-KEY (*test**) OR TITLE-ABS-KEY (*diagnos**) OR TITLE-ABS-KEY (*"death rate"*) OR TITLE-ABS-KEY (*complication**))) AND (LIMIT-TO (DOCTYPE , *"ar"*) OR LIMIT-TO (DOCTYPE , *"sh"*) OR LIMIT-TO (DOCTYPE , *"cp"*)) AND (LIMIT-TO (LANGUAGE , *"English"*))	1,826

ISI / WOS

N		Queries	Results
1		**TOPIC:** ("2019 novel coronavirus") *OR* **TOPIC:** ("COVID19") *OR* **TOPIC:** ("COVID-19") *OR* **TOPIC:** ("COVID 2019") *OR* **TOPIC:** ("2019-novel CoV") *OR* **TOPIC:** ("SARS-cov-2") *OR* **TOPIC:** ("SARS-CoV2") *OR* **TOPIC:** ("SARSCoV2") *OR* **TOPIC:** ("SARSCoV-2") *OR* **TOPIC:** ("severe acute respiratory syndrome coronavirus 2") *OR* **TOPIC:** ("2019-ncov") *OR* **TOPIC:** ("coronavirus disease 2019") *OR* **TOPIC:** ("coronavirus disease-19") *OR* **TOPIC:** ("2019ncov") *OR* **TOPIC:** ("SARS coronavirus 2")	49,428
2		**TOPIC:** (ethnic*) *OR* **TOPIC:** (disparit*) *OR* **TOPIC:** (race*) *OR* **TOPIC:** (minorit*) *OR* **TOPIC:** (inequit*) *OR* **TOPIC:** (socioeconomic*) *OR* **TOPIC:** (poverty inequalit*) *OR* **TOPIC:** ("social determinants of health") *OR* **TOPIC:** (indigence*) *OR* **TOPIC:** (discrimination) *OR* **TOPIC:** (equit*) *OR* **TOPIC:** (income) *OR* **TOPIC:** ("social class")	1,135,220
3		**TOPIC:** (access*) *OR* **TOPIC:** (hospitalization*) *OR* **TOPIC:** (mortality*) *OR* **TOPIC:** (morbidity*) *OR* **TOPIC:** ("diagnostic tests") *OR* **TOPIC:** ("new case*") *OR* **TOPIC:** ("basic reproduction number") *OR* **TOPIC:** ("intensive care") *OR* **TOPIC:** ("prevalence") *OR* **TOPIC:** ("incidence") *OR* **TOPIC:** ("case fatality rate") *OR* **TOPIC:** (test*) *OR* **TOPIC:** (diagnos*) *OR* **TOPIC:** ("death rate") *OR* **TOPIC:** (complication*)	9,437,320
4		#3 AND #2 AND #1	1,134
5	#3 AND #2 AND #1	You searched for: (#3 AND #2 AND #1) *AND* **LANGUAGE:** (English)<br>**Timespan:** 2019-2020. **Indexes:** SCI-EXPANDED, SSCI.	1,119

Embase

N		Queries	Results
#1		'2019 novel coronavirus':ti,ab,kw OR 'covid19':ti,ab,kw OR 'covid-19':ti,ab,kw OR 'covid 2019':ti,ab,kw OR '2019-novel cov':ti,ab,kw OR 'sars-cov-2':ti,ab,kw OR 'sars-cov2':ti,ab,kw OR 'sarscov2':ti,ab,kw OR 'severe acute respiratory syndrome coronavirus 2':ti,ab,kw OR 'coronavirus disease 2019':ti,ab,kw OR '2019ncov':ti,ab,kw OR 'coronavirus disease 2019':lnk	70,053
#2		ethnic*:ti,ab,kw OR disparit*:ti,ab,kw OR 'health status disparities':lnk OR 'healthcare disparities':lnk OR race*:ti,ab,kw OR race:lnk OR minorit*:ti,ab,kw OR 'minority health':lnk OR racism:lnk OR inequit*:ti,ab,kw OR socioeconomic*:ti,ab,kw OR poverty:ti,ab,kw OR 'socioeconomic factors':lnk OR 'social class':lnk OR 'social class':ti,ab,kw OR inequalit*:ti,ab,kw OR 'health equity':lnk OR poverty:lnk OR 'social determinants of health':ti,ab,kw OR 'social determinants of health':lnk OR indigence*:ti,ab,kw OR discrimination:ti,ab,kw OR equit*:ti,ab,kw OR income:ti,ab,kw OR indigence:lnk OR 'population characteristics':lnk OR 'ethnic group':lnk	934,416
#3		access*:ti,ab,kw OR 'health care access':lnk OR hospitalization*:ti,ab,kw OR hospitalization:lnk OR mortality*:ti,ab,kw OR mortality:lnk OR morbidity*:ti,ab,kw OR morbidity:lnk OR 'diagnostic tests':ti,ab,kw OR 'new case*':ti,ab,kw OR 'basic reproduction number':ti,ab,kw OR 'basic reproduction number':lnk OR 'intensive care':ti,ab,kw OR 'prevalence':ti,ab,kw OR 'incidence':ti,ab,kw OR 'incidence':lnk OR 'case fatality rate':ti,ab,kw OR 'case fatality rate':lnk OR test*:ti,ab,kw OR diagnos*:ti,ab,kw OR 'death rate':ti,ab,kw OR complication*:ti,ab,kw	10,725,007
#4	#1 AND #2 AND #3		1,759
#5	#1 AND #2 AND #3	#1 AND #2 AND #3 AND ([article]/lim OR [article in press]/lim OR [conference abstract]/lim OR [conference paper]/lim OR [data papers]/lim OR [short survey]/lim) AND [english]/lim AND [2019-2020]/py	1,455
