# Supplementary material for: A systematic review of racial/ethnic and socioeconomic disparities in COVID-19
Source: Int J Equity Health. 2021 Nov 24;20:248. doi: 10.1186/s12939-021-01582-4 (PMC8611382; doi:10.1186/s12939-021-01582-4)
Supplement: Supplementary file 2 — Additional file 2:. Data extraction table. [file 12939_2021_1582_MOESM2_ESM.docx]

Additional file 2. Data Extraction Table

| **Raw** | **Author/ year** | **Country/ setting** | **Type of study;** | **Race/ ethnicity identification** | **Participants/ Sample size** | **Data source** | **Data analysis** | **Exposure** | **Outcome measures** | **Results** |
| --- | --- | --- | --- | --- | --- | --- | --- | --- | --- | --- |
| 1 | (Abedi et al., 2020) | US  369 counties from seven most affected states (Michigan, New York, New Jersey, Pennsylvania, California, Louisiana, Massachusetts | Ecological study county-level | self-reported national  and state demographic data from from USA facts and the US Census Bureau | N= 102,178,117 population  Black (n=718)  Asian (n=695)  White (n=738)  Hispanic (n=696) | (1) Publicly available data from USA facts and the US Census Bureau for COVID-19 cases and county-level demographic data  (2) COVID-19 data reported by each state on their department of health websites,  (3) State Population by race/ethnicity data,  (4) Mobility data extracted from Google. | Correlation, and regression were used to test hypotheses of association | Black  Asian  Hispanic  Uninsured  Poverty | Rate of  COVID-19 infection and all COVID-19-related death as provided by each state’s department of health as of April 09, 2020. | Risk of COVID-19 Infection   - Counties with a higher population (est. 0.34, 95% CI 0.24, 0.44, q < 1.1E-08), a higher median income (est. 0.36, 95% CI 0.25, 0.48, q < 2.3E-08), and a more diverse population (higher percentage of Hispanics, Asians, and Blacks) have a higher rate of infection. - A higher percentage of Asians (est. 0.32, 95% CI 0.20, 0.44 q < 6.3E-07, women; est. 0.32, 95% CI 0.20, 0.43, q < 4.5E-07, men), Blacks (est. 0.47, 95% CI 0.32, 0.62, q < 2.3E-08, women; est. 0.35, 95% CI 0.20, 0.51, q < 1.7E-05, men), and Hispanics (est. 0.49, 95% CI 0.34, 0.64, q < 1.2E-08, women; est. 0.46, CI 0.31, 0.62, q < 8.1E- 08, men) are associated with a higher rate of infection while a higher percentage of non-Hispanic Whites (est. − 0.41, 95% CI − 0.55, − 0.26, q < 2.9E-07, women; est. − 0.44, 95% CI −0.58, − 0.30, q < 4.2E-08, men) is associated with a lower rate of COVID-19. - Change in grocery mobility (est. − 0.24, 95% CI − 0.36, − 0.13, q < 5.5E-05), retail mobility (est. − 0.26, 95% CI − 0.38, − 0.14, q < 4.5E-05), and work mobility (est. − 0.31, 95% CI − 0.43, − 0.20, q < 9.0E-07) were associated with a lower rate of infection. - Higher percentage of disability (est. − 0.159, 95% CI − 0.265, − 0.053, q < 0.006). - With respect to their percentage of uninsured and found no significant association other than among men (est. 0.181, 95% CI 0.05, 0.313, q < 1.2E-02) and non- Hispanic Whites (est. 0.251, 95% CI 0.123, 0.380, q < 3.1E- 04). - in our stepwise regression model, minorities specifically Black and Hispanic women, poverty, and level of education among non-Hispanic Whites, disability, the total county population, and level of mobility are predictors of the rate of COVID-19 infection.   Rate of Mortality   - Protective factors for the counties are a higher percentage of Asians (est. − 0.27, 95% CI − 0.41, − 0.12, q < 0.003, women; est. − 0.23, 95% CI − 0.37, − 0.09, q < 0.009, men) and education level with a bachelor’s degree or higher with an odds ratio ranging from − 0.41 to − 0.03 across the various ethnicities. Other protective factors for counties include having a higher percentage of people insured (strongest indicator being for non-Hispanic White people with an estimate of− 0.48, 95% CI − 0.68, − 0.28, q < 6.0E-05) and median income (est. − 0.27, 95%CI − 0.41, − 0.12, q < 0.003). - The total population in the counties is also a major indicator (est. − 0.33, 95% CI − 0.43, − 0.20, q < 6.0E-05) of lower COVID-related death. - Factors significantly associated with higher mortality in the counties analyzed include a higher percentage of people under the poverty level (for all the races analyzed in this study), a higher percentage of people on Medicaid (est. 0.17, 95% CI 0.03, 0.30, q < 0.04), and a higher rate of people with disability in the county (est. 0.27, 95%CI 0.09, 0.45, q < 0.02). Grocery mobility was also highly associated with mortality (est. 0.21, 95% CI 0.02, 0.39, q < 0.06).   COVID-19 Infection and Mortality Are Higher Among African Americans   - The number of African Americans infected by COVID-19 is 64,605 (1981 cases per million) with the number of deaths reaching 6181 (211 deaths per million), while the number of Whites, as defined in the reports, is 104,914 (658 cases per million) infected and 9806 (76 deaths per million) dead leading to a disproportional percentage of African Americans infected (p < 0.0001) by COVID-19 and dead (p < 0.0001) as the result. The number of infected Latinos and Asians per million, as defined in the reports, is 947 and 390, while the rate of mortality (per million) is 82 and 52 respectively. |
| 2 | (Adegunsoye et al., 2020) | US;  at the university of chicago. | Retrospective cohort analysis | Electronic medical records | N=4,413 patients  who underwent nasopharyngeal swab and sars-cov-2 polymerase chain reaction assays after clinical screening (january 1, 2020 to april, 15, 2020)  Black n= 2,543 (58%)  White n= 1,071 (24%)  Other n=799 (18%) | (1) The most recent electronic medical records.  (2) Self-service cohort discovery tool (see cohorts) from the center for research informatics. | Two-sided t tests, Mann–Whitney U tests, or chi-square tests  Logistic regression models | Black | Covid-19 infection  Covid-19 mortality  Covid-19 hospitalization | Mortality   - There were no observed sex or racial differences in mortality among all SARS-CoV-2–positive patients in the entire cohort (P=0.48 and P = 0.34, respectively).   Odds of infection   - Analyses using univariate logistic regression models demonstrated that Black race was associated with SARSCoV-2 infection (odds ratio [OR], 3.30; 95% confidence interval [95% CI], 2.75–3.97) and hospitalization (OR, 3.77; 95% CI, 2.38–5.99) but not mortality. These results remained consistent in multivariable logistic regression models (OR, 2.16; 95% CI, 1.73–2.70 and OR, 1.51; 95% CI, 1.03–1.05, respectively   Age-adjusted SARS-CoV-2 infection rates in Black and non-Black patients.   - The SARS-CoV-2 infection rate was 10-fold higher among subjects aged 30–50 years than for those aged 0–18 years (0.05 vs. 0.005). The age-adjusted SARS-CoV-2 positivity rate (0.14) remained higher in Black individuals compared with non-Black individuals (0.19 vs. 0.07). |
| 3 | (Adhikari et al., 2020) | US  Urban US counties at 10 major us cities, all of which experienced early surges of covid-19 infections. | Cross-sectional | United States Census Bureau | N= 158 counties | 1) 2018 us census small area income and poverty estimates program.  2) Publicly available by the centers for disease control and prevention as well as state health departments | Random-effect linear regression  models | Minority (not specified): categorizing counties as Substantially White, Less diverse, More diverse, and Substantially non-White  Poverty | Cumulative incidence (total number of COVID-19 infections and deaths per 100 000  individuals) | - In more-poverty counties, those with substantially non-White populations had an infection rate nearly 8 times that of counties with substantially White populations (RR, 7.8; 95%CI, 5.1-12.0) and a death rate more than 9 times greater (RR, 9.3; 95%CI, 4.7-18.4). - we found that among both more-poverty and less-poverty counties, those with substantially non-White or more diverse populations had higher expected cumulative COVID-19 incident infections compared with counties with substantially White or less-diverse populations (eg, more diverse counties with less poverty: RR, 3.2; 95%CI, 2.3-4.6). Similar associations were observed for deaths (eg, more diverse counties with less poverty: RR, 3.8; 95%CI, 2.2-6.7). |
| 4 | (Ahmad et al., 2020) | US  3135 US counties | cross sectional ecological analysis | US Census Bureau | N= 3135 US counties total population mean±SD= 98418.71 ± 313273.6  (82–9818605) | County-level data from the US Centers for Disease Control, US Census Bureau and John Hopkins Coronavirus Resource Center for 3135 US counties. | Multilevel generalized linear models with a negative binomial distribution family and a log link function | Percentage of households in a county with poor housing condition | Incidence rate ratios (IRR) (cases/100,000)  mortality  rate ratios (MRR) (death/100,000) | - In the adjusted models standardized by county population, with each 5% increase in percent households with poor housing conditions, there was a 50% higher risk of COVID-19 incidence (IRR 1.50, 95% CI: 1.38–1.62) and a 42% higher risk of COVID-19 mortality (MRR 1.42, 95% CI: 1.25–1.61). |
| 5 | (Aldridge et al., 2020) | England | Prospective cohort | NHS data on patients with a positive COVID-19 test | N= 16,272 deaths from COVID-19  Asian n= 1,354 (8%)  Black n= 989 (6%)  Mixed n= 126 (0%)  Other n= 485 (2%)  White n= 13,317 (81%) | NHS data on patients with a positive COVID-19 test  who died in hospitals in England published on 28th April | Exact Poisson  method | Minority ethnic group: Black African, Black Caribbean, Pakistani, Bangladeshi and Indian. | Death from COVID-19 | - The largest total number of deaths in minority ethnic groups were Indian (492 deaths) and Black Caribbean (460 deaths) groups. - Adjusting for region, we found a lower risk of death for White Irish (SMR 0.52; 95%CIs 0.45-0.60) and White British ethnic groups (0.88; 95%CIs 0.86-0.0.89), but increased risk of death for Black African (3.24; 95%CIs 2.90-3.62), Black Caribbean (2.21; 95%CIs 2.02-2.41), Pakistani (3.29; 95%CIs 2.96-3.64), Bangladeshi (2.41; 95%CIs 1.98-2.91) and Indian (1.70; 95%CIs 1.56-1.85) minority ethnic groups. |
| 6 | (Azar et al., 2020) | US  Sutter  Health, a large not-for-profit integrated health  system in northern California | Retrospective cohort | Patient self-reported data collected by Sutter Health. | Group1 N= 14,036 patients  who were tested for COVID-19 from January 1–  April 8, 2020  Group2 N= 1,052 confirmed cases of  COVID-19 By April 8  Group1:  Non-Hispanic White n= (48.3%)  Asian n= (10.2 %)  African American n= (6.7%)  American Indian/Pacific Islander n= (0.7%)  Hispanic n= (19.1%)  Other n= (15.0 %)  Group2:  Non-Hispanic White n= (40.7%)  Asian n= (11.8 %)  African American n= (5.8%)  American Indian/Pacific Islander n= (0.2%)  Hispanic n= (25.8%)  Other n= (15.8%) | Electronic health record (ehr) data from sutter health. | t-tests for  continuous variables and chi-square tests for  Logistic regression | African American ethnicity  Household income (median income level by Census ZIP Code Tabulation  Areas (ZCTAs)  categorized by quartiles.)  Type of insurance | Testing  Hospital admission | - A smaller percentage of African Americans (29.9 percent) were tested for COVID-19 in an ambulatory setting compared to whites (56.0 percent), Asians (60.0 percent), and Hispanics (53.8percent) (exhibit2). The majority of African Americans were tested in hospitals, either in the ED(37.8 percent) or as inpatients (32.3 percent). - The likelihood of hospital admission for African Americans was more than double that of whites (OR = 2.7; p ¼ 0:007). - People with Medicaid or who were self-pay or had no reported insurance had twice the odds of being admitted, compared to those with commercial insurance (OR = 2.1 for both; p < 0:05). - COVID-19 positive patients residing in ZIP codes within the top two quartiles of income (quartiles 3 and 4) were less likely to be admitted to the hospital than those residing in the bottom quartile ZIP code (OR = 0.24 and 0.55 for the top two quartiles). |
| 7 | (Baqui et al., 2020) | Brazil  central-south region and north region | A cross-sectional observational study | Self-reported data on IVEP-Gripe public  Dataset. | N= 11 321 patients | SIVEP-Gripe (Sistema de Informação de Vigilância Epidemiológica da Gripe) dataset. | Mixed-effects Cox regression survival analysis | Black ethnicity | Mortality | - Compared with White Brazilians, Pardo and Black Brazilians admitted to hospital had significantly higher risk of mortality (HR 1·45, 95% CI 1·33–1·58 for Pardo Brazilians; 1·32, 1·15–1·52 for Black Brazilians). - Notably, Pardo ethnicity was the second most important risk factor for death after age. |
| 8 | (de Lusignan, Dorward, et al., 2020) | UK | Cross-sectional | Medical record | N= 3802 SARS-CoV-2 test results  White n= 2497 (65.7%)  Asian n= 152 (4.0%)  Black n= 58 (1.5%)  Mixed, other n= 81 (2.1%)  Missing 1014 (26.7%) | Computerized primary care medical records of sentinel practices | Univariate logistic regression models  Multivariate logistic regression models | Black ethnicity  Urban | Confirmed positive test | - Compared with white people, the adjusted odds of a positive test were greater in black people (388 [15·5%] of 2497 white people vs 36 [62·1%] of 58 black people; adjusted OR 4·75, 95% CI 2·65–8·51). - People living in urban areas versus rural areas (476 [26·2%] of 1816 in urban areas vs 111 [5·6%] of 1986 in rural areas; adjusted OR 4·59, 95% CI 3·57–5·90) and in more deprived areas (197 [29·5%] of 668 in most deprived vs 143 [7·7%] of 1855 in least deprived; adjusted OR 2·03, 95% CI 1·51–2·71) were more likely to test positive. |
| 9 | (Drefahl et al., 2020) | Sweden | Population-based cohort study | - | N= 7,775,064 individuals with an average follow-up time of 56 days amounting to a total of 1,189,484 person-years under observation. | individual-level Swedish register data collected and maintained at various state agencies, and combined and stored for research purposes at statistic sweden’s secure data storage facility | Multivariate Cox regression | Lower education  Lower income | Covid-19 deaths | - In working ages, those in the lowest income tertile are more than five times as likely to die (HR: 5.40; 95% CI: 3.51, 8.35) from COVID-19 than those in the highest tertile. - In working ages, those with primary (HR: 2.62; 95% CI: 1.65, 4.16) and secondary (HR: 2.22; 95% CI: 1.46, 3.37) education are more than twice as likely to die relative to those with postsecondary education. - Immigrants from low and middleincome countries are approximately twice as likely to die, as compared to individuals born in Sweden. |
| 10 | (Egede et al., 2020) | US  Milwaukee and  Southeast Wisconsin | Cross-sectional | Self-report in the medical record | N=31,549 adults tested for  COVID-19 between March 1 and July 10, 2020  Non-Hispanic White (75.4%)  Non-Hispanic Black (19.8%)  Hispanic (4.8%) | Froedtert/Medical  College of Wisconsin (MCW) Health System’s  Epic electronic medical record (last updated July  10, 2020) | Analysisof-  variance, chi-square, and Fisher’s exact tests  Unadjusted and adjusted logistic regression  and Cox proportional hazard models | Non-Hispanic Black  Hispanic | Confirmed cases  Hospitalization  Mortality | - In adjusted analyses, Blacks were 3.7 times more likely (adjusted odds ratio: 3.65; 95% CI: 3.23, 4.13) and Hispanics were 3.1 times more likely (AOR: 3.07; 95% CI: 2.57, 3.66) to have a positive COVID-19 test compared with Whites. - Among those who had a positive COVID-19 test, members of minority groups were two times more likely to be hospitalized (for Blacks, AOR: 2.15,95%CI: 1.56, 2.98; and for Hispanics, OR: 2.03, 95% CI: 1.25, 3.28) compared with Whites after adjustment for demographics and comorbidities. - After adjustment, compared with Whites, Hispanics were two times more likely to die (AHR, 2.09; 95% CI: 1.04, 4.16), and there was a small but statistically nonsignificant difference for Blacks (AHR: 1.11; 95% CI: 0.68, 1.82) |
| 11 | (Farrell et al., 2020) | Ireland  an Irish hospital | Cross-sectional | Emergency Department’s Symphony electronic  information software system | N=257 patients admitted with a diagnosis of SARS-CoV-2 infection  White–Irish n = 164 (63.8%)  White–other n = 44 (17.1%)  BAME n = 49 (24.1%) | Microbiology  Department generated and maintained a Microsoft Excel  spreadsheet database detailing all confirmed cases of SARSCoV-2 infection collected by our hospital’s laboratory. Other information were recorded. | Multivariable regression | Deprivation  Non-Irish White  Black, Asian or minority ethnic (BAME) | Death  ICU admittance | Death   - Deprivation was a strong predictor of mortality, even after adjustment for age, with a percentage point increase in deprivation associated with a 5% increase in mortality (adjusted HR [95% CI] = 1.05 [1.01, 1.09], p = 0.012). - Patients from care homes were more likely (adjusted HR [95% CI] = 2.68 [1.24, 5.60], p = 0.012) to die than community patients after adjusting for age. - Compared with White Irish people, those of other White (p = 0.886) and BAME (p = 0.405) ethnicities did not have an increased risk of hospital death, after adjusting for age. This was also the case when ethnicity was broken down into further subcategories.   ICU admittance   - After adjusting for age, deprivation (p = 0.196) was not associated with admittance to ICU. - Compared with White Irish patients, all other ethnic groups including other White (4.22 (1.45, 12.31); p = 0.008) and other BAME (4.58 (1.33, 15.84); p = 0.018) had an approximately fourfold increased risk of ICU admittance after adjusting for age. |
| 12 | (Goyal et al., 2020) | US  An exclusively pediatric drive-through and walk-up SARS-CoV-2 testing site | Cross-sectional study | Online referral form included | N= 1000 children (0 and  22 years) tested for SARS-CoV-2 infection  non-Hispanic (NH)-white n= 203 (20.3%)  NH-black n= 304 (30.4%)  Hispanic n= 229 (22.9%)  Other n= 171 (17.1%)  Unknown n= 93 (9.3%) | census block group from the  American Community Survey  (2014–2018) | bivariable and multivariable logistic regression | Minority ethnic groups: non-Hispanic Black, Hispanic  Low family income  Q4: [$157,679->$250,000]  Q3: [$107,321-$157,308]  Q2: [$70,341-$107,292]  Q1: [$11,667-$70,300] | rates of infection | - In comparison with non-Hispanic white children (7.3%), minority children had higher rates of infection (non-Hispanic Black: 30.0%, adjusted odds ratio [aOR] 2.3 [95% confidence interval (CI) 1.2–4.4]; Hispanic: 46.4%, aOR 6.3 [95% CI 3.3–11.9]). - In comparison with children in the highest median family income quartile (8.7%), infection rates were higher among children in quartile 3 (23.7%; aOR 2.6 [95% CI 1.4–4.9]), quartile 2 (27.1%; aOR 2.3 [95% CI 1.2–4.3]), and quartile 1 (37.7%; aOR 2.4 [95% CI 1.3–4.6]). |
| 13 | (Gu et al., 2020) | US  at the University of Michigan | Retrospective cohort study | Self-reported data from electronic health records (EHRs) | N= 5698 patients tested or treated for COVID-19 at the University of Michigan from March 10, 2020, to  April 22, 2020.  White n= 3740 (65.6%)  Black n= 1058 (18.6%)  Other n= 544 (9.5%)  Unknown n= 356 (6.2%) | National Neighborhood Data Archive, a publicly available  data source;  electronic health records (EHRs) | Logistic regression | Black ethnic group  Populated living area (Population density,  persons/square mile) | Hospitalization  in ICU admission  mortality | - Adjusting for age, sex, socioeconomic status, and comorbidity score, Black patients were more likely to be hospitalized compared with White patients (OR, 1.72 [95%CI, 1.15-2.58]; *P* = .009). - In addition to older age, male sex, and obesity, living in densely populated areas was associated with increased risk of hospitalization (OR, 1.10 [95%CI, 1.01-1.19]; P = .02). - No statistically significant racial differences were found in ICU admission and mortality based on adjusted analysis. |
| 14 | (D. Hawkins, 2020) | US  Massachusetts | Ecological study | - | N= 87 256 cases of COVID-19 diagnosed in MA through June 10, 2020 | 5-year estimates (2014-2018) from the American Community Survey (ACS) in Massachusetts (MA). | Poisson regression with a log-link | Lower social determinants of health (variables: Percentage of residents in poverty, Median income, Employment in healthcare and social assistance industry, Employment in transportation industry, Employment in service occupations, Employment in healthcare support occupations, Percentage of residents who rented, Percentage of residents who were uninsured, Unemployment rate) | rates of COVID-19  Testing  Positive test | - Cities and towns with a higher percentage of residents living in poverty and lower median incomes tended to have elevated rates of COVID-19. - With respect to employment, cities and towns with more workers employed in the healthcare and social assistance and transportation industries and in service and healthcare support occupations also tended to have high­er rates of COVID 19. - Communities with a higher proportion of their population renting and uninsured had ele­vated COVID-19 rates. - Cities and towns with higher levels of poverty, renting, and lack of insurance, lower median incomes, and higher employment in the transportation industry and service and healthcare support occupations tended to have a higher percentage of positive tests. - Most of these differences were not accounted for by different rates of testing in these cities and towns. |
| 15 | (Holmes et al., 2020) | US  Selected states based on disparity in the previous epidemics | Cross-sectional ecologic design | State department of health | N= about five states  % of population  Blacks/AA 13% | State department of health that provide COVID-19  cases and mortality by gender, county or location, age and race | Proportion: percent change of outcome measures by race  Binomial regression model (BRM) | Black African American (Black/AA) | Cumulative  incidence  Case fatality | Cumulative incidence   - While Blacks/AA represent 13% of the population of the US as per US Census projection (2020), overall mortality was 34% (n = 19,062), with a 21% excess cumulative incidence of dying as per the 04/09/20 data with a total death of 5700.   Case fatality rate   - Case fatality for Blac/AA was 2.7%, while the case fatality for White was 2.5%, X^2^ (4) =13.6, p = 0.001 (9th April). - The COVID-19 CF was higher among Blacks/AA relative to Whites; Maryland, (2.7% vs. 2.5%), Wisconsin (7.4% vs. 4.8%), Illinois (4.8% vs. 4.2%), Chicago (5.9% vs. 3.2%), Detroit (Michigan), 7.2% and St. John the Baptist Parish (Louisiana), 7.9% Blacks/AA compared to Whites in Michigan were 15% more likely to die, CmI risk ratio (CmIRR) = 1.15, 95% CI, 1.01–1.32. - Blacks/AA relative to Whites in Illinois were 13% more likely to die, CmIRR = 1.13, 95% CI, 0.93–1.39, while Blacks/AA compared to Whites in Wisconsin were 51% more likely to die, CmIRR = 1.51, 95% CI, 1.10–2.10. In Chicago, Blacks/AA were more than twice as likely to die, CmIRR = 2.24, 95% CI, 1.36–3.88. |
| 16 | (Holtgrave et al., 2020) | US  New York State | Continuum construction | CDC National Center for Health  Statistics Bridged Race File,  Vintage 2018. | N=13,990,900 adults 18 years or older of NY  Non-Hispanic white n= 8,968,100 (64%)  Non-Hispanic Black n= 2,287,400 (16%)  Hispanic n= 2,735,400 (19%) | CDC National Center for Health Statistics Bridged Race File, Vintage 2018.  New York State Department of  Health antibody seroprevalence Study.  New York City Department of  Health and Mental Hygiene  COVID-19 data report.  Retrospective cohort study of hospitalized New York COVID-19 patients.  Online archive of officially reported New York State  COVID-19 hospitalizations and Deaths. | Descriptive analysis  Proportion (Ratio versus white, non-Hispanic) | Black non-  Hispanic, and Hispanic adults | Overall fatality per population (“PFR”)  Race-specific  infection fatality rates (per infection-experienced individuals,  “IFR”)  Case-fatality rates (per diagnosed persons,  “CFR”)  Hospitalization | - Compared with white non-Hispanic adults, racial/ethnic minority populations had disproportionately higher per-population likelihoods of COVID-19 diagnosis (0.93% white non-Hispanic, 1.89% Black non-Hispanic, 1.85% Hispanic), hospitalization (0.11% white non-Hispanic, 0.50% Black non-Hispanic, 0.48% Hispanic), and death (0.03% white non-Hispanic, 0.18% Black non-Hispanic, 0.12% Hispanic). - Among individuals with infection experience, diagnosis rates varied by race and ethnicity: 11.7% of infection-experienced white non-Hispanic adults were diagnosed compared with 10.1% of Black non-Hispanic adults and 6.5% of Hispanic adults. - Levels of hospitalization among persons diagnosed were about two-fold higher Black non-Hispanic and Hispanic adults compared with white non-Hispanic adults, with hospitalizations also relatively elevated among persons who were infected. - The population fatality rate ratios illustrate that per population, Black non-Hispanic and Hispanic adults were, respectively, 5.38 and 3.48 times as likely to die of COVID-19 as were white non-Hispanic adults. - Conditioning among those who were infected, the IFR for Black non-Hispanic adults remained 2.30-fold that of white non-Hispanic, whereas no disparity remained for Hispanic versus white non-Hispanic adults (IFR ratio = 0.98). - Among those diagnosed, the relative CFR was 2.65 for Black non-Hispanic versus white non-Hispanic adults, and 1.75 for Hispanic versus white non-Hispanic adults.   Summarized   - Estimated per-population COVID-19 fatality rates were 0.03%, 0.18%, and 0.12% for white non-Hispanic, Black non-Hispanic, and Hispanic adults, respectively. - The 3.48-fold disparity for Hispanic, relative to white, communities was explained by differences in infection experience, whereas the 5.38-fold disparity for non-Hispanic Black, relative to white, communities was primarily driven by differences in both infection experience and in the need for hospitalization, given infection. |
| 17 | (Ioannou et al., 2020) | US  Department of Veterans Affairs (VA) national  health care system | Longitudinal cohort | VA Informatics and  Computing Infrastructure (VINCI) | N= 88,747 patients  tested for SARS-CoV-2 nucleic acid by polymerase chain reaction between Feburary 28 and May 14.  White n= 5,022 (49.6%)  Black n= 4,215 (41.6%)  Asian n= 80 (0.8%)  American Indian or American Native, Native Hawaiian or Pacific Islander n= 140 (1.4%)  Unknown n= 674 (6.7%) | VA’S corporate data warehouse, a data repository of electronic medical records, developed by the VA informatics and computing infrastructure (VINCI) to facilitate research. | Cox proportional hazards models | Black ethnicity | Risk of hospitalization, mechanical ventilation, and death  Positivity | - Compared with individuals who tested negative, those testing positive were more likely to be Black individuals (19 340 [24.6%] vs 4215 [41.6%]). - Compared with White patients, Black patients were more likely to be hospitalized (aHR, 1.13; 95%CI, 1.04-1.23) and to receive mechanical ventilation (aHR, 1.52; 95%CI, 1.25-1.85) but no more likely to die (aHR, 1.04; 95%CI, 0.88-1.21). - Areas with high regional COVID-19 disease burden were associated with increased risk of death (eg, ≥700 vs <130 deaths per 1 million residents: aHR, 1.21; 95%CI, 1.02-1.45). - Summarized:   Compared with white non-Hispanic adults, Black non-Hispanic populations had 0.5% and Hispanic populations had 0.37% higher per-population likelihoods of COVID-19 hospitalization.  Compared with white non-Hispanic adults, Black non-Hispanic populations had 0.15% and Hispanic populations had 0.09% higher per-population likelihoods of COVID-19 death |
| 18 | (Joseph et al., 2020) | US  Urban quaternary-care academic  medical center with affiliated community health centers that serve catchment areas with a relatively high proportion of individuals  of Hispanic ethnicity and high-density housing according  to census data | Single-institution retrospective cohort study | Self-reported  information  available in the electronic medical record | N= 326  Patients hospitalized  with confirmed COVID-19 infection between March 17, 2020, and April 10, 2020.  White (non-Hispanic) n= 116 (35.6%)  Black n= 27 (8%)  Asian n= 10 (3%)  Hispanic n= 142 (43.6%)  Other/unavailable n= 31 (9.5%) | Electronic medical record (Epic Hyperspace; Epic Systems, Verona, Wis) | Multivariable linear regression analyses | non-White (ie, Hispanic,  Black, Asian, or other) | Severity of lung disease on admission chest radiographs (using the modified Radiographic Assessment of Lung  Edema (mRALE) score)  Secondary outcome was a composite adverse clinical outcome of intubation, intensive care unit admission,  or death | - Non-White patients had significantly higher mRALE scores (median score, 6.1; 95% confidence interval [CI]: 5.4, 6.7) compared with White or non-Hispanic patients (median score, 4.2; 95% CI: 3.6, 4.9) (unadjusted average difference, 1.8; 95% CI: 0.9, 2.8; P < .01). - Multivariable linear regression analyses demonstrated that non-White patients presented with higher mRALE scores at admission chest radiography compared with White or non-Hispanic patients (adjusted average difference, 1.6; 95% CI: 0.5, 2.7; P < .01). - Adjustment for hypothesized mediators revealed that the association between race and/or ethnicity and mRALE scores was mediated by limited English proficiency (P < .01).   Key points   - Non-White patients admitted with confirmed coronavirus disease 2019 (COVID-19) infection were more likely to have increased disease severity at admission chest radiography, as measured by using the modified Radiographic Assessment of Lung Edema (mRALE) score (adjusted average difference, 1.6; P < .01). - Higher mRALE scores were associated with an increased likelihood of adverse composite clinical outcomes for White or non-Hispanic patients (adjusted hazard ratio, 1.3; P < .001) and non-White patients (adjusted hazard ratio, 1.2; P < .001). - Increased radiographic severity among non-White patients was associated with delayed presentation, low English proficiency, and obesity. - Among White or non-Hispanic patients (n = 116), 90 (78%) required supplemental oxygen, 36 (31%) were admitted to the intensive care unit, 29 (25%) were intubated, and 27 (23%) died. Among non- White patients (n = 210), 153 (73%) required supplemental oxygen, 83 (40%) were admitted to the intensive care unit, 72 (34%) were intubated, and 19 (9%) died |
| 19 | (Kabarriti et al., 2020) | US  Montefiore Medical Center in New York | Cohort study | Self-reported information in the electronic medical record | N= 5,902 with positive test results of 9268 patients tested for COVID-19 between March 14 and April 15, 2020.  Non-Hispanic White n= 509 (8.6%)  Non-Hispanic Black n= 1935 (32.8%)  Hispanic n= 1905 (32.3%)  Asian n= 171 (2.9%)  Other n= 792 (13.4%)  Unknown n= 590 (10.0%) | Electronic medical record | χ2 tests,  Univariable and multivariable Cox proportional hazards regression | Hispanic and non-Hispanic Black | Case fatality rates  Survival | - Hispanic and non-Hispanic Black patients were also more likely to test positive for COVID-19 than White patients, with 1905 of 2919 Hispanic patients (65.3%), 1935 of 2823 non-Hispanic Black patients (68.5%), and 509 of 960 non-Hispanic White patients (53.0%) having positive test results for SARS-CoV-2 (P < .001). - While controlling for age, sex, socioeconomic status and comorbidities, patients identifying as Hispanic (hazard ratio, 0.77; 95%CI, 0.61-0.98; P = .03) or non-Hispanic Black (hazard ratio, 0.69; 95%CI, 0.55-0.87; P = .002) had slightly improved survival compared with non-Hispanic White patients. |
| 20 | (Kaufman et al., 2020) | US  all 50 states and the District of Columbia | Cohort study | Patient zip code data as reported by the 2018 5-year American  Community Survey (ACS) on the US Census data website | N= 2,331,175 patients with positive SARSCoV-  2 NAAT test result.  Black non-  Hispanic (12.4%)  Hispanic (22.1%) White non-Hispanic (56.1%)  Asian non-Hispanic (5.9%)  Other (3.5%) | Informatics Data  Warehouse;  2018 5-year American  Community Survey (ACS) on the US Census data website. | Cochran-Armitage test  Multivariable logistic regression  model | Black non-Hispanic community and Hispanic community | Trends of confirmed cases (positivity) | - Analysis of 2,331,175 unique patients tested March-May 2020 demonstrated an increasing trend in SARS-CoV-2 NAAT positivity across Black non-Hispanic community progressive quintiles (from 7.8% to 17.2%, P < 0.0001) and Hispanic community progressive quintiles (from 8.4% to 15.5%, P < 0.0001) and a decreasing trend across White non-Hispanic community progressive quintiles (from 17.4% to 7.1%, P < 0.0001). - Statistically significant increasing trends in positivity for the Black non-Hispanic, Hispanic, and Asian non-Hispanic populations were demonstrated in both the highest and lowest income areas (P< 0.0001 for all). - Statistically significant decreasing trends in positivity for the White non-Hispanic population and the ‘‘other’’ race/ethnicity populations were demonstrated in both the highest and lowest income areas (P < 0.0001 for all). - Statistically significant increasing trends in positivity for the Black non-Hispanic and Hispanic populations were demonstrated in both the most densely populated areas (P < 0.0001 for all) and in the least densely populated areas (P < 0.0001 for all). - Statistically significant decreasing trends in positivity for the White non-Hispanic population and the ‘‘other’’ race/ethnicity populations were demonstrated in the most and least densely populated areas (P < 0.0001 for both). - Statistically significant decreasing trends in positivity for the Asian non-Hispanic population were demonstrated in the most densely populated areas (P < 0.0001) but there was no significant trend in the least densely populated areas. |
| 21 | (Khan et al., 2020) | England,  Glasgow, Lanarkshire | Prospective cohort study | - | N=172 hospitalized patients  with confirmed COVID‐19. | Electronic case notes | The Student t test | Low Socioeconomic status (SES)  Scottish index for multiple deprivation (SIMD) was used to divide patients into two groups: more deprived (SIMD 1‐5) and less deprived (SIMD 6‐10) | Need for intubation  Death | - The median time to discharge was 7 days longer (17 vs 10 days; P = .018) and median time to death was 4.5 days longer (17 vs 12.5 days; P = .388) in more deprived group. - When comparing SIMD 1 to SMID 10, the rate of hospitalization was similar (0.03% vs 0.03%; P = .926). - There is no statistically significant difference in both groups for transfer to critical care, intubation, 30‐day all‐cause mortality, and overall poor outcome. |
| 22 | (Kim et al., 2020) | US  Three hospitals and more than 300 clinics  across the Puget Sound region | Cross-sectional study | - | N= 562 242 living patients who had at least 1 encounter  (including telephone or telemedicine) in our system from January 1, 2019, to February 28, 2020  non-English speakers 1869 (6.0%) | Registration data and laboratory test | Descriptive comparison | Non-English speakers | Positive test  Completed testing | - Non-English speakers were overall less likely to have completed testing compared with English-speakers (4.7% [95%CI, 4.5%-4.9%] vs 5.6%[95%CI, 5.6%-5.7%]) - Notably, the proportion of positive cases was 4.6-fold higher among non-English speakers overall (18.6%; 95%CI, 16.8%-20.4%) compared with English speakers (4.0%; 95%CI, 3.8%-4.2%) |
| 23 | (Lassale et al., 2020) | England | Community-based cohort study | self-reported UK Biobank, | N= 428,494 participants  White  Asian or Asian  British (7.3%)  Black or Black British (7.8%)  Other (Chinese, Mixed and Other) | UK Biobank study | Logistic regression models | Ethnic minority groups | Risk of COVID-19  Hospitalization | - Compared to the White study members and after adjusting for age and sex, Black individuals had over a 4-fold increased risk of COVID-19 infection (odds ratio; 95% confidence interval: 4.32; 3.00–6.23), and there was a doubling of risk in the Asian group (2.12; 1.37, 3.28) and the ‘other’ non-white group (1.84; 1.13, 2.99). - After controlling for potential explanatory factors which included neighbourhood deprivation, household crowding, smoking, body size, inflammation, glycated haemoglobin, and mental illness, these effect estimates were attenuated by 33% for Blacks, 52% for Asians and 43% for Other, but remained raised for Blacks (2.66; 1.82, 3.91), Asian (1.43; 0.91, 2.26) and other non-white groups (1.41; 0.87, 2.31). |
| 24 | (Mahajan & Larkins-Pettigrew, 2020) | US  National | Correlation analysis | New York Times dataset | N=2886 counties | - Datasets on COVID-19 confirmed cases and deaths by county from the publicly available New York Times GitHub repository datasets on racial and population totals by county from the Centers for Disease Control and Prevention website | Pearson product-moment correlations (percentage of COVID-19 confirmed cases and deaths by  dividing the totals reported in the New York Times dataset by the total number of individuals in the respective county) | African–Americans  Asian–Americans | Conformed cases  Death  Case mortality (number who die from COVID-19 divided by number who are infected in that county) | - A positive correlation existed between percentages of African–Americans living in a county and who have COVID-19 (r = 0.254, P < 0.0001), who have died from COVID-19 (r = 0.268, P < 0.0001), and case mortality (r = 0.055, P = 0.003). - There was also a positive correlation between percentage of Asian–Americans living in a county and percentage who have COVID-19 in that county (*r* = 0.185, *P <* 0.0001) and percentage who have died from COVID-19 in that county (*r* = 0.211, *P <* 0.0001). - There was a negative correlation between percentage of Whites living in a county and percentage who have COVID-19 in that county (r =−0.287, P <0.0001) and percentage who have died from COVID-19 in that county (r = −0.308, P < 0.0001). - There were weak correlations between racial percentages living in a county and COVID-19 case mortality rates in those counties (number who die from COVID-19 divided by number who are infected in that county) (*r* = 0.055 and *P* = 0.003, *r* = 0.026 and *P* = 0.168 and *r* = −0.0479 and *P* = 0.010 for African–Americans, Asian–Americans and Whites, respectively). |
| 25 | (Misa et al., 2020) | US  Northern California  Alameda Health System (AHS) | Retrospective cohort study | Electronic health record | N= 526 patients tested for COVID-19.  Black n= 214 (40.7%)  Latinx n= 139 (26.4%)  White n= 83 (15.8%)  Asian n= 41 (7.8%)  Other n= 14 (2.7%)  Unknown n= 35 (6.7%) | Electronic health record (EHR) and  stored it in REDCap (a secure online data collection instrument). | Multivariable logistic regression | Latinx  Black | Test positivity rate  30 day in-hospital mortality | Test positivity   - The test positivity rate among Latinx patients was 40.3% (56/139) compared with 10.1% (39/387) among non-Latinx patients (p < 0.001). Latinx ethnicity was associated with COVID-19 test positivity (adjusted odds ratio 9.6, 95% confidence interval: 3.5–26.0). - The test positivity rate among Black patients was 7.5% (16/214) compared with the test positivity rate of 25.3% (79/312) among non-Black patients (p< 0.001). The test positivity rate among white patients was 9.6% (8/83) compared with a test positivity rate of 19.6% (87/443) among non-white patients (P = 0.03). - The test positivity rate among Asian patients was 14.3% (6/42) compared with the test positivity rate of 18.4% (89/484) among non-Asian patients (p < 0.51). - Latinx ethnicity, unknown race or ethnicity, lack of primary care physicians, and skilled nursing facility residence were associated with higher odds of COVID-19 test positivity. Latinx patients had an adjusted odds ratio (aOR) of 9.6 (95% CI: 3.5–26.0; predictive margin 0.39); unknown race or ethnicity had an aOR of 4.5 (95% CI: 1.3–15.7; predictive margin 0.26); lack of a primary care physician had an aOR of 2.0 (95% CI: 1.1–3.4, predictive margin 0.23); skilled nursing facility residence had an aOR of 10.6 (4.6–24.6; predictive margin 0.46) of COVID-19 test positivity.   Mortality   - Mortality among Black patients was higher than non-Black patients (18.7% vs 1.3%, p < 0.001). - The test positivity rate among patients with Spanish as their primary language was 46.8% (52/111) compared with the test positivity rate of 10.4% (43/415) among non-Spanish speakers. |
| 26 | (Munoz-Price et al., 2020) | US  Froedtert Health and Medical College  of Wisconsin (Milwaukee), the largest academic system in Wisconsin | Cross-sectional study | self-reported data available in the electronic medical record | N= 2595 consecutive  adults tested for COVID-19 from March 12 to March 31, 2020.  African American n= 785 (30.2%)  White n= 1617 (62.3%)  Other n= 193 (7.4%) | Electronic medical record | Multivariable, generalized estimating equations (GEE) logit models | Black  Poverty status (ie, uninsured or  receiving Medicaid) | COVID-19 positivity,  Hospitalization  Intensive care  unit admission,  Mechanical  ventilation  Death | Positive tests   - Regardless of SES, African American patients were considerably more likely to test positive for the virus than persons of other races (odds ratio [OR], 5.37; 95%CI, 3.94-7.29; P = .001), even after controlling for differences in demographic and health characteristics, comorbidities, presenting symptoms, and clustering at the zip-code level. - Poverty status was not significant (OR = 0.66; CI = 0.41-1.08; *P* = .10).   hospitalization   - Adjusting for zip code of residence, Black race (OR, 1.85; 95%CI, 1.00-3.65; *P* = .04) and poverty (OR, 3.84; 95%CI, 1.20-12.30; *P* = .02) were associated with hospitalization.   intensive care admission/ ventilation/ mortality   - Poverty (OR, 3.58; 95% CI, 1.08-11.80; *P* = .04) but not Black race (OR, 1.52; 95%CI, 0.75-3.07; *P* = .24) was associated with intensive care unit admission. - There were no statistically significant differences between African American patients and patients from other racial groups in ICU admission (44 [20.2%] vs 26 [17.2%]; P = .47), mechanical ventilation (15 [6.9%] vs 11 [7.3%]; P = .88), or in-hospital mortality among those admitted (14 [17.9%] vs 6 [15.9%]; P = .62) - Results for mechanical ventilation and death indicated that neither race nor poverty were significantly associated with these outcomes.   Other   - African American patients testing positive for COVID-19, compared with patients who belonged to other racial groups, were more likely to be younger (≥60 years, 78 of 218 [35.8%] vs 70 of 151 [46.4%]; P = .04), to have no insurance or receive Medicaid (81 [37.1%] vs 18 [11.9%]; P = .001), to have 3 or more comorbidities (60 [27.5%] vs 25 [16.5%]; P = .03). |
| 27 | (Niedzwiedz et al., 2020) | UK | prospective cohort  study | Self-reported derived from the baseline assessment  centre data collection | N= 2658 participants who  attended UK Biobank baseline assessment centres in  England.  White British n= 348,735 (88.9)  White Irish n= 9800 (2.5)  White Other n= 12,925 (3.3)  Mixed n= 2356 (0.6)  Indian n= 4571 (1.2)  Pakistani n= 1259 (0.3)  Other South Asian n= 1493 (0.4)  Black Caribbean n= 3669 (0.9)  Black African n= 2623 (0.7)  Black Other n= 103 (0.0)  Chinese n= 1153 (0.3)  Others n= 3429 (0.9) | UK Biobank;  Public Health England (PHE) microbiology database Second  Generation Surveillance System and linked to UK  Biobank baseline data. | Poisson regression with robust standard errors | Black and south Asian groups  Deprivation:  Quartile 1 (most advantaged) n= 100,701 (25.7)  Quartile n= 2 99,838 (25.5)  Quartile n= 3 98,380 (25.1)  Quartile 4 (least advantaged) n= 93,197 (23.8) | Test positivity | - Black and south Asian groups were more likely to test positive (RR 3.35 (95% CI 2.48–4.53) and RR 2.42 (95% CI 1.75–3.36) respectively), with Pakistani ethnicity at highest risk within the south Asian group (RR 3.24 (95% CI 1.73–6.07)). - Socioeconomic deprivation was associated with a higher risk of confirmed infection (RR 2.19 for most deprived quartile vs least (95% CI 1.80–2.66). |
| 28 | (Ojinnaka et al., 2020) | US  Texas counties | Retrospective study (county-level) | 2020 Robert Wood Johnson  Foundation (RWJF) County Health Rankings data. | N= 254 Texas counties  Mean ± SD  Percent non-Hispanic white 55.39 ± 21.04  Percent Black 6.26 ± 6.40  Percent Asian 1.33 ± 2.08  Percent Hispanic 35.35 ± 22.99 | Texas Department of State Health  Services and merged with the 2020 Robert Wood Johnson foundation (RWJF) county health rankings data | Logistic regression  model | African Americans/Blacks  Hispanics  Unemployment | COVID-19 death;  Cumulative COVID-19 ;  fatalities per  100,000 population; | - The odds of having a COVID-19 fatality decreased with a unit increase in the rate of primary care physicians in a county (OR = 0.93; 95% CI = 0.89, 0.99). - In the unadjusted model, there was a statistically significant increase in COVID-19 deaths/100,000 population with every 1% increase in the proportion of African Americans/Blacks (β = 6.04; 95% CI = 0.94, 11.14), non-Hispanic Whites (β = 5.38; 95% CI = 0.21, 10.34), and Hispanics (β = 5.63; 95% CI = 0.67, 10.59). - We observed a significant increase in COVID-19 deaths with every 1 % increase in the proportion of African Americans/Blacks (β = 5.08; 95% CI = 0.11, 10.04) and Hispanics (β = 5.41; 95% CI = 0.52, 10.31), but not for non-Hispanic whites or Asians.   socioeconomic   - In addition, there was a significant increase in COVID-19 deaths/100,000 with every 1 % increase in the proportion of adults with obesity (β = 0.71; 95% CI = 0.12, 1.30), unemployed individuals (β = 5.21; 95% CI = 2.22, 8.20) and in primary care physician rate (β = 0.17; 95% CI = 0.02, 0.32). |
| 30 | (Renelus et al., 2020) | US  New York City (NYC);  university-affiliated NYC hospital | Single-center retrospective cohort study | Electronic health record | N= 734 patients with COVID-19  Blacks n= 372 (50.7%),  Whites n= 214 (29.2%), Hispanics n= 92 (12.5%),  Asians n= 20 (2.7%)  Others n = 36 (4.9) | Electronic health record; NYC Department of Health data | Chi-square and t tests;  Cox proportional hazards regression | Black  Hispanics  Asian | Hospitalization;  In-hospital mortality; | - Blacks were nearly twice as likely as Whites to require hospitalization for COVID-19 (OR 1.89, 95%CI, 1.59–2.24, p < 0.001). - There was no statistically significant difference in odds for COVID-19 hospitalization between Hispanics and Whites (OR 0.84, 95% CI 0.66–1.07, p = 0.15). - After adjusting for age and the other variables, Hispanics had a statistically significant increased hazard of in-hospital mortality (HR 1.84; 95% CI 1.21–2.80; p= 0.005) along with Asians (HR 2.06; 95% CI, 1.08–3.93; p= 0.03). - There was a non-significant increased hazard of in-hospital mortality among Blacks when compared with Whites (HR, 1.30; 95% CI, 0.95–1.78; p= 0.09). |
| 31 | (Rentsch et al., 2020) | US  Veterans Affairs | Retrospective cohort study | Documented race/ ethnicity | N=5,834,543 individuals in clinical care (defined as having at least 1 clinical encounter between January  1, 2018, and December 31, 2019, and alive as of January 1, 2020)  White n= 4,309,613 (73.9%)  Black n= 1,089,883 (18.7%)  Hispanic n= 435,047 (7.5%) | Electronic health record database of the Department of Veterans Affairs (VA) | Logistic regression  models | Black  Hispanics | Receipt of COVID-19 testing;  Test positivity;  30-day mortality; | - Black individuals were more likely to be tested (rate per 1,000 individuals: 60.0, 95% CI 59.6–60.5) than Hispanic (52.7, 95% CI 52.1–53.4) and White individuals (38.6, 95% CI 38.4–38.7). - Individuals from minority backgrounds were more likely to test positive (Black versus White: odds ratio [OR] 1.93, 95% CI 1.85–2.01, p < 0.001; Hispanic versus White: OR 1.84, 95% CI 1.74–1.94, p < 0.001). - 30-day mortality did not differ by race/ethnicity (Black versus White: OR 0.97, 95% CI 0.80–1.17, p = 0.74; Hispanic versus White: OR 0.99, 95% CI 0.73–1.34, p = 0.94). |
| 32 | (Rodriguez et al., 2020) | US  88 hospitals in the American Heart Association (AHA) COVID-19 CVD Registry | Retrospective data analysis | Zip code | N= 8,950 patients hospitalized with COVID-19 from 1/17/2020 to 7/22/2020.  non-Hispanic White  (59.3%),  Black (10.6%), Hispanic (all races) (9%),  Asian (4.7%). | American Heart Association COVID-19 Cardiovascular Disease Registry | sequential multivariable logistic regression | Black ethnicity  Hispanics ethnicity  Asian ethnicity | in-hospital mortality; | - The adjusted odds ratios (ORs) for mortality were 0.93 (95% confidence interval [CI] 0.76-1.14) for Black patients, 0.90 (95% CI 0.73-1.11) for Hispanic patients, and 1.31 (95% CI 0.96-1.80) for Asian patients compared with non-Hispanic White patients. The median OR across hospitals was 1.99 (95% CI 1.74-2.48). - Although in-hospital mortality and MACE did not differ by race/ethnicity after adjustment, Black and Hispanic patients bore a greater burden of mortality and morbidity due to their disproportionate representation among COVID-19 hospitalizations. |
| 33 | (Sapey et al., 2020) | UK  University Hospitals Birmingham  NHS Foundation Trust (UHB) in Birmingham | Retrospective cohort study | Self- reported Ethnicity data for the Birmingham and Solihull area from  the 2011 census | N= 2 217 patients with COVID-19  White n= 1540 (69.5) Mixed/multiple n= 18 (0.8) South Asian/South Asian British n= 410 (18.5) Black/African/Caribbean/black British n= 134 (6.0) Other ethnic group n= 67 (3.0) Preferred not to say n= 22 (1.0) Not known n= 26 (1.2) | electronic healthcare record (EHR) and mandatory data sets within the  Trust. | Cox regression | Asian ethnicity  Black | Death while in hospital or  post discharge | - South Asian ethnicity was associated with an increased risk of death, both by Cox regression (HR 1.4, 95% CI 1.2 to 1.8), after adjusting for age, sex, deprivation and comorbidities, and by propensity score matching, matching for the same factors but categorising ethnicity into South Asian or not (HR 1.3, 95% CI 1.0 to 1.6). - No significant difference was reported in adjusted model for Black ethnic groups. |
| 34 | (Soares et al., 2020) | Brazil  Esp´ırito Santo state | cohort of SARSCoV-  2–infected patients | Patents' documentary | N= 10,713 patients with COVID-19  White n= 3,748 (35%)  Black/multiracial n= 4,513 (42.1%)  Asian/indigenous/unknown n= 2,452 (22.9%) | State Health Secretariat of Esp´ırito Santo, Brazil; on June 11, 2020, last updated on June 11. | Multivariable logistic regression models. | Asian ethnic groups;  Black/multiracial groups | Hospitalization  COVID-19 death in hospitalized patients | - Asian ethnic groups were at higher risk of hospitalization compared to Withes OR: 1.47 (1.23–1.75, < 0.001). - No significant association were found regarding the risk of hospitalization of Black/multiracial groups. - No significant association was found between race/ethnicity of any group and COVID-19 death in hospitalized patients. |
| 35 | (Yehia et al., 2020) | US  92 hospitals in 12 states: Alabama (6 hospitals), Maryland (1  hospital), Florida (5 hospitals), Illinois (8 hospitals), Indiana (14 hospitals), Kansas (4 hospitals),  Michigan (13 hospitals), New York (2 hospitals), Oklahoma (6 hospitals), Tennessee (4 hospitals),  Texas (11 hospitals), and Wisconsin (18 hospitals). | Cohort study | Self-reported data from electronic health records and administrative data | N= 11 210 adult patients hospitalized with confirmed severe acute respiratory coronavirus 2 (SARS-CoV-2) between February 19, 2020, and May 31, 2020, in 92 hospitals in 12 states.  White n = 4,606 (41%)  Black n = 4,180 (37.3%)  With/other/ missing n = 2,424 (21.7%) | Electronic health records and administrative data. | Cox proportional  hazards regression with mixed effects | Black ethnic groups  Unknown or Medicare insurance (insurance categorized as Commercial, Mdeicare, Medicaid, other, unknown) | Death during hospitalization | - After adjustment for age, sex, insurance, comorbidities, neighborhood deprivation, and site of care, there was no statistically significant difference in risk of mortality between Black and White patients (hazard ratio, 0.93; 95%CI, 0.80 to 1.09). - Patients with Medicare insurance (HR, 1.47; 95%CI, 1.08-2.00) and individuals whose insurance coverage was unknown (HR, 2.17; 95%CI, 1.32-3.57) had higher risk of mortality than those with commercial insurance. |
| 36 | (Zakeri et al., 2020) | UK  King’s College  Hospital Foundation Trust (KCHFT), which comprises two separate hospitals in south London. | Case-control and a cohort study | electronic health record (EHR) | n = consecutive adult patients  (age ≥18 years) requiring emergency hospital admission with a primary  diagnosis of COVID-19, between 1 March and 2 June 2020.  872 cases group; n=3,488 control group. | electronic health record (EHR);  Primary healthcare database (Lambeth DataNet). 1 March and 2 June 2020. | conditional logistic regression  models | Black (African, Caribbean, any other Black);  Asian (Indian,  Pakistani, Bangladeshi, Chinese, any other Asian), | Admission risk;  Inhospital mortality | - Black and Mixed/Other ethnicity were associated with higher admission risk than white (OR 3.12 [95% CI 2.63-3.71] and 2.97 [2.30-3.85] respectively). Adjustment for comorbidities and deprivation modestly attenuated the association (OR 2.24 [1.83_2.74] for Black, 2.70 [2.03_3.59] for Mixed/Other). - Asian ethnicity was not associated with higher admission risk (adjusted OR 1.01 [0.70_1.46]). - Age and male sex, but not Black (adjusted HR 1.06 [0.821.37]) or Mixed/Other ethnicity (adjusted HR 0.72 [0.47-1.10]), were associated with in-hospital mortality. - Asian ethnicity was associated with higher inhospital mortality but with a large confidence interval (adjusted HR 1.71 [1.15-2.56]). |
| 37 | (Lieberman-Cribbin et al., 2020) | US  New York City (NYC) | Retrospective observational | Obtained from 2018 American Community Survey  5-year estimates. | 177 ZIP code Tabulation  Areas (ZCTA) in NYC | the NYC Coronavirus (COVID-19) data repository hosted by the NYC Department of Health from March 2, 2020 current through April 6,2020. | Multivariable Poisson regressions | Hispanic composition  SES index: median household income in the past 12  months (Table B19013), median gross rent, percentage  living <150% of the poverty line (e.g., an annual salary of  ≤$26,200 for a 4-person household), education, percentage working class, percentage  Unemployed, and more than 1 occupant per  Room. | Test positivity  The number of total tests | - The number of total tests significantly increased with the increasing proportion of white residents (B= 0.004, SE=0.001, p=0.0032) but not with increasing Hispanic composition or SES index score. - The ratio of positive tests to total tests significantly decreased with the increasing pro-portion of white residents in the ZIP code Tabulation Area (B= - 0.003, SE=0.000 6, p<0.001) and with increasing SES index score (B= - 0.001 6, SE=0.0007, p=0.0159). - The ratio of positive tests to total tests significantly decreased with the increasing proportion of white residents in the ZCTA (B= - 0.003, SE=0.0006, p<0.001) and the increasing SES index score (B=-0.0016, SE=0.0007, p=0.0159). |
| 38 | (Loomba et al., 2021) | US | Retrospective study (state-level) | US Census Bureau | N= 50 states | Absolute counts for COVID-19 cases, tests and mortality were obtained from Worldomete from March 13th, 2020 through April 30th,2020. | Univariate and multivariate regression | Black  Asian  Insurance | Mortality  Testing  Frequency  COVID-19 case frequency | - Lower prevalence of uninsured (beta coefficient - 298.6, p-.01 were associated with greater case frequency on univariate analysis. - lower prevalence of uninsured (beta coefficient - 900.9, p=.02) were associated with greater testing frequency. - lower prevalence of uninsured (beta coefficient - 0.1, p=.02) were associated with greater percent mortality on univariate linear regression analyses. - No significant association were found between higher frequency of Black or Asian residents and case frequency, testing, and mortality on univariate analysis. |
| 39 | (Ayoubkhani et al., 2020) | England and  Wales  usual residents of England and  Wales enumerated in private households | Retrospective cohort study | self-reported data from 2011 Census | N=47 872 412 residents  White (86.4%)  Bangladeshi and Pakistani (3.0%)  Black (3.2%)  Chinese (0.6%)  Indian (2.6%)  Mixed (2.1%)  Other (2.2%) | 2011 Census to the 2011–2013 NHS  Patient Registers | Cox-proportional-hazard models  hazard  ratios (HRs) | Black  Bangladeshi/Pakistani, Indian, Mixed and Other ethnic backgrounds | Mortality (age-standardized mortality rates (ASMRs)) | - For both males and females, the ASMRs of COVID-19 mortality were greatest among individuals identifying as Black (250.6 and 116.9 deaths per 100 000 people, respectively) and lowest among those identifying as White (87.3 and 51.8 deaths per 100 000 people, respectiveely). - age-adjusted HRs indicated that males and females from all ethnic-minority groups (except females of Chinese ethnicity) were at greater risk of death involving COVID-19 compared with those of White ethnicity. The rate of death involving COVID-19 was 3.13 (95% confidence interval [CI]: 2.93–3.34) times greater for Black males than for White males, and 2.40 [2.20–2.61] times greater for Black females than White females. - People of Bangladeshi/Pakistani, Indian, Mixed and Other ethnic backgrounds also had raised rates of death involving COVID-19 compared with those of White ethnicity. |
| 40 | (Baena-Diez et al., 2020) | Spain  Barcelona | Ecological study  (district level) | - | N= 10 districts of the city of  Barcelona | Official COVID-19  registry of the Catalan Government’s Department of Health;  26 February 2020 and 19 April 2020 | Spearman rho | Poverty (lower income districts) | COVID-19 incidence | - The lower the mean income, the higher the COVID-19 incidence (Spearman rho = 0.83; P value = 0.003). - Districts with the lowest mean income had the highest incidence of COVID-19 per 10 000 inhabitants; in contrast, those with the highest income had the lowest incidence. - Specifically, the district with the lowest income had 2.5 times greater incidence of the disease, compared with the highest-income district [70 (95% confidence interval 66–73) versus 28 (25–31), respectively]. |
| 41 | (Boserup et al., 2020) | US  48 states/regions | cross-sectional study (state-level data) | self-reported data from US Census Bureau American  Community Survey 5-Year Estimate | N= 173 counties  spanning 37 states from  March 1, 2020, to July 11, 2020. | COVID-19 Tracking Project and the Centers for Disease Control and Prevention (CDC). Demographic data were obtained from the US Census Bureau, social vulnerability data were obtained from the CDC, social distancing data were obtained from Unacast, and medical disparities data from the Center for Medicare and Medicaid Services. | Negative binomial regression | Deprivation;  Unemployment;  English proficiency | COVID-19 death rates | Deprivation   - The predicted number of white COVID-19 deaths/100 000 population was significantly higher in counties with an increased percentage of households without a vehicle (Incidence Rate Ratio (IRR) = 1.04, 95% CI: 1.02- 1.05, P < .001) and significantly higher in counties with an increased percentage of white Medicare beneficiaries with diabetes (IRR = 1.07, 95% CI: 1.02-1.12, P = .004). - The predicted number of black COVID-19 deaths/100 000 population was significantly higher in counties with an increased percentage of households without a vehicle (IRR = 1.05, 95% CI: 1.03-1.08, P < .001) and significantly higher in counties with an increased percentage of black Medicare beneficiaries with diabetes (IRR = 1.12, 95% CI: 1.04-1.20, P = .002). Conversely, the predicted number of black COVID-19 deaths/100 000 population was significantly lower in counties that had better social distancing scores (IRR = .48, 95% CI: .24-.94, P = .033) and significantly lower in counties with higher unemployment rates (IRR = .84, 95% CI: .73-.97, P = .020). - The predicted number of Hispanic COVID-19 deaths/ 100 000 population was significantly higher in counties with an increased percentage of persons (age ≥5 years) who speak English “less than well” (IRR = 1.22, 95% CI: 1.10-1.34, P < .001), was significantly higher in counties with an increased percentage of households with no vehicle available (IRR = 1.03, 95% CI: 1.004-1.052, P = .022), and was significantly higher in counties with an increased percentage of Hispanic Medicare beneficiaries with diabetes (IRR = 1.18, 95% CI: 1.11-1.25, P < .001). Additionally, the predicted number of Hispanic COVID-19 deaths/100 000 population was significantly lower in counties with higher unemployment rates (IRR = .86, 95% CI: .76-.98, P < .022) and was significantly lower in counties with an increased percentage of Hispanic Medicare beneficiaries with COPD (IRR = .89, 95% CI: .81-.98, P = .023). |
| 42 | (DiMaggio et al., 2020) | US  New York | Ecological study | self-reported data from U.S. Census | N= 177 ZIP code Tabulation  Area (ZCTAs)  Proportion black 0.23  Proportion hispanic 0.12 (0.05) | The New York City  Department of Health and Mental Hygiene (NYC DOHMH) GitHub  Page; April 3e22, 2020 | Bayesian hierarchical Poisson spatial models | Black/African American residents  Household income  Housing density | Test positivity | - There was a nearly five-fold increase in the risk of a positive COVID-19 test (incidence density ratio ¼ 4.8, 95% credible interval 2.4, 9.7) associated with the proportion of black/African American residents. - For each unit increase in a standardized measure of median household income in a ZCTA, there is an approximately 46% decrease in the number of positive COVID-19 tests (IDR = 0.54; 95% Cr I: 0.43, 0.69). - Increases in the proportion of residents older than 65 years, housing density, and the proportion of residents with heart disease were each associated with an approximate doubling of risk. - Proportion of persons not speaking English, and the proportion of persons on public assistance were not associated with positive COVID-19 testing rates. |
| 43 | (Fielding-Miller et al., 2020) | US  all 50 states | Observational (county-level) | self-reported data from US  census Bureau | N=3024 counties from all 50 states | County public health agencies, aggregated and made publicly available by the New York Times (NYT). as of July 12, 2020.  American Community Survey’s (ACS) 2014 5-year estimate.  US Bureau of Economic Analysis.  US Census Small  Area Health Insurance Estimates (SAHIE) program’s 2018 estimates. | Spatial autoregressive models. | Poverty  Uninsured  Non-English  Language  Farm worker occupation | COVID-19 mortality | - The percentage of non-English speaking households in a county was significantly associated with higher rates of death across all counties in New England (total b = 2.9, p = 0.02), the Mid-Atlantic (total b = 4.0, p < 0.001), and West North Central states (total b = 1.5, p < 0.001) and in non-urban counties in Mountain states (b = 1.20, p = 0.02). - The percentage of uninsured individuals was associated with fewer reported COVID-19 deaths per 100,000 residents across all counties in New England (b = -3.6, p = 0.04) and in non-urban New England counties (b = -3.1, p = 0.02), but with higher rates across all counties in the Mid-Atlantic (b = 6.8, p = 0.01). - Poverty was associated with 4.2 fewer reported deaths per 100,000 residents across all Mid-Atlantic counties (p <0.001) and 3.7 fewer reported COVID-19 deaths per 100,000 residents in non-urban Mid-Atlantic counties (p = 0.002), but with 4 more reported deaths per 100,000 residents in all counties in the East South Central region (p = 0.02) and 3.7 more reported deaths per 100,000 residents in non-urban counties in the same region (p = 0.03). |
| 44 | (Figueiredo et al., 2020) | Brazil | Ecological study | - | N= all Brazilian Federative Units (FU) | The Brazilian Ministry of Health regarding  the number of confirmed cases and deaths by COVID-19 until  August 23, 2020 | Multiple  linear regression | Household income;  Overcrowded households;  Unemployment; | Incidence  Mortality | - Incidence of COVID-19 included three independent variables as predictors, those being the Gini Index of household income (β = 0.365; t = 2.355; p = 0.027), household overcrowding (β = 0.353); t = 2.289; p = 0.032) and the lethality rate (β = - 0.489; t = -3.630; p = 0.001) generating a model with better adjusted R2 and better level of statistical significance [F (3.23) = 111.387; p < 0.001; R2 = 0.598]. - Regarding the dependent variable “mortality”, the final model also included the independent variables Gini Index (β = 0.407; t = 2.567; p = 0.017) and overcrowding (β = 0.352; t = 2.236; p = 0.017); and lethality rate (β = 0.351; t = 2.565; p = 0.035). This way, it was generated a model with adjusted R2 and better statistical significance level [F (3.23) = 10.528; p < 0.001; R2 = 0.579]. |
| 45 | (R. B. Hawkins et al., 2020) | US  All 50 states | Cohort study | self-reported data from American Communities Survey 5-year Estimates and Census Bureau County and Zip Code Business Patterns | N= 3,127 counties with COVID-19 data available. | Publicly available dataset of aggregated county sources (USAFacts) on May 2, 2020.  Centers for Disease Control suggest strong racial disparities in both COVID-19 prevalence and outcomes.  The 2018 Behavioral Risk Factor Surveillance System (BRFSS) and U.S. Census population data | Hierarchical linear mixed models. | Black residents  Education level | Confirmed cases (100,000 population)  Fatality (100,000 population) | - The median percentage of black Americans was significantly higher in severely distressed counties compared with less distressed counties (7.3% vs 1.8%, p<0.0001). - The median percentage of uninsured individuals was significantly higher in severely distressed counties compared with less distressed counties (14.9% vs 10.0%, p<0.0001). - A higher number of cases were associated with lower education level, higher proportion of black Americans, higher income and lower poverty rate. The specific covariates with significant associations with cases/100,000 persons were percentage of adults without a high school degree (RR 1.10), proportion of black residents (RR 1.03), median income ratio (RR 1.01), and poverty rate (RR 0.98). - Higher COVID-19 mortality was associated with higher income but lower education, higher employment rate, higher proportion of black Americans, older residents, and less obesity. The significant socioeconomic associations were percentage of adults without a high school degree (RR 1.08), median income ratio (RR 1.01), and percentage of adults not working (RR 0.98). Significant demographic and health related associations included percentage of population over 65 (RR 1.07), proportion of black residents (RR 1.03), and obesity prevalence (RR 0.96). |
| 46 | (Hu et al., 2020) | US  city/town level in Massachusetts | Observational | self-reported data from U.S. Census Bureau | N= 6,547,785 people of Massachusetts  Non-Hispanic Black (2.75%)  Hispanic (4.83%)  Asian (3.60%)  Non-Hispanic White (91.06%) | Department of Public Health in Massachusetts.  The subcounty and census block group (CBG) 2018 American Community Survey  (ACS).  Google Maps Distance  Matrix API. | Two classical spatial autoregressive  models, including the spatial lag model (SLM) and the spatial error model (SEM), | Hispanic  and Non-Hispanic Black/African Americans  Poverty  Overcrowding house holds (high housing densities) | COVID-19 incidence  Testing site access | - With a parameter estimate of -66.217 (SEM), the rate of the population below the poverty level had a significantly negative influence on the COVID-19 incidence rate. - The results of the three models demonstrated that income inequality had a nonsignificant and negative impact on the COVID-19 incidence rate. - The result of SEM indicated that a one-point increase in the rate of households with more than 1 occupant per room was associated with a 157.385-point increase in COVID-19 incidence rate. - Non-Hispanic Black had the lowest weighted travel time of 5.69 min to the testing sites, followed by the Hispanic, Asian, and White groups. Access to hospitals showed similar results, with a minor deference. - The Hispanic group surpassed the Non-Hispanic Black with the lowest travel time of 8.88 min. Compared with the other three groups, the White group had the longest travel times of 7.82 and 11.21 min to testing sites and hospitals, respectively, which is consistent with the lowest COVID-19 incidence rate in more concentrated quantiles. |
| 47 | (Madhav et al., 2020) | US  Neighborhoods in Louisiana | Observational | - | N= 4,138 population (64 parishes (counties)  and 1,148 census) | American  Community Survey (ACS) 2018.  Louisiana Department of Health website on July 31, 2020. | Poisson regression analyses | Deprivation (based on Area Deprivation Index (ADI) Quintiles (Q)): Quintile 1 is the reference group and refers to the least deprived neighborhoods and quintile 5 refers to the  most deprived neighborhoods. | Rate of COVID-19 infection | - In the crude model (model 1) the most deprived neighborhoods (5th quintile) had a 30% higher rate of COVID-19 infection compared to those in the least deprived (1st quintile) neighborhoods (RR = 1.30, 95% CI = 1.17–1.39). - In model 2, adjusted for the effect of urban residence, those living in the most deprived neighborhood (5th quintile) had a 39% higher rate of COVID-19 infection compared to those living in the least deprived neighborhood (1st quintile), after adjusting for urban/rural location. - Although urban location was also significantly associated with COVID-19 infection (RR 1.32, 95% CI 1.22–1.43), there was no substantial effect of urban location on the relationship between ADI quintiles and COVID-19 infection (interaction term estimate: 0.0010, p = 0.4729, data not shown). |
| 48 | (Raine et al., 2020) | US  national | Cross-sectional | Self-reported national  and state demographic data from the United States Census Bureau’s 2018 American Community  Survey (ACS) 5 year averages | US population; 45 out of the 50 US states  White 61.10%  Latinx 17.80%  Black 12.30%  Asian 5.40%  Multiracial 2.40%  American Indian or Alaskan Native (AIAN) 0.70%  Native Hawaiian or Pacific Islander (NHPI) 0.2%  Unknown 0.2% | COVID Boston University’s  Center for Antiracist Research COVID Racial Data Tracker.  American Community Survey (ACS).  1March and 14 June 2020 | A representation quotient (RQ) was utilized;  An RQ greater than 1 indicates that a subgroup is over represented, and a RQ less than 1 indicates that the subgroup is under-represented.  The magnitude of the RQ can be used to evaluate the scale of the under- or over-representation | Non-White, non-Black Hispanic/Latinx | Incidence  Mortality | - The case RQ for White people was only 0.484. This indicates that, for White people, COVID-19 case representation is approximately 48% of the group’s representation in the total US population. - Non-White, non-Black Hispanic/Latinx accounted for 25.58% of the confirmed cases in the US and had a RQ of 1.437. This indicates that the representation of Hispanic/Latinx in COVID-19 cases was 43% greater than the group’s representation in the US population. AIAN, NHPI, and Black people all had RQs >1; 1.303, 1.115, and 1.278, respectively. The case RQ for Asian people was 0.509. - White people accounted for the largest proportion of confirmed mortality by race, approximately 49.4% of total US deaths. However, the case RQ for White people was only 0.808. This indicates that the White population’s representation in COVID-19 deaths is 80.8% of the White representation in the total US population. - Black people accounted for only 22.04% of the total number of confirmed deaths in the US, but had a RQ of 1.792. indicating that Black people account for a larger portion of COVID-19 deaths relative to their representation in the US population. Asian, AIAN, and NHPI people each had RQs less than 1; 0.742, 0.829, and 0.352, respectively |
| 49 | (Ossimetha et al., 2021) | US  All counties with at least one SARS-CoV-2 case by May 15, 2020 | Observational | - | N= 2664 counties | Reported on April 1, 2020 (baseline level for this study), and May 15, 2020, were obtained from a repository maintained by the Johns Hopkins University Center of Systems Science and Engineerin. | Locally weighted scatter plot smoothing (LOESS).  Linear regression  with state-fixed effects | Lower social deprivation index (SDI) | SARS-CoV-2 cases/1000 population  Death | - In analyses adjusted for SARS-CoV-2 prevalence on April 1, medium- and high-SDI counties had 1.39 (95% CI, 0.85 to 1.93; P<.001) and 2.56 (95% CI, 1.77 to 3.34; P<.001) more SARS-CoV-2 cases/1000 population on May 15 compared with low-SDI counties, respectively. Deaths per capita were also significantly higher for higher-SDI counties. |
| 50 | (Khanijahani & Tomassoni, 2021) | US  all 50 statesand the District of Columbia | Retrospective observational | Self-reported data from U.S. Census Bureau. | N= 3142 county (73,056 population)  Mean (SD)  % in Black-concentrated 13.3 (27.2) | the Center for Systems Science and Engineering (CSSE) at Johns Hopkins University; from January 22 to July 21, 2020. | mixed-effect negative binomial regression | Black population (Black-concentrated neighborhoods if 25% or more of the res-idents were Black.)  Disadvantaged area | Confirmed COVID-19 deaths per 100,000 population | - For every 10% increase in the percentage of county population residing in concentrated disadvantage and Black-concentratedtracts, the rate for confirmed COVID-19 deaths per 100,000 population increases by a factor of 1.14 (mortality rate ratio [MMR] =1.14; 95% confidence interval [CI]:1.11, 1.18) and 1.11 (MMR = 1.11; 95% CI:1.08, 1.14), respectively. |
| 51 | (Khanijahani, 2021) | US  3,142 counties in 50 states and the District of Columbia | Retrospective observational | Self-reported data from American Community Survey (ACS) 5-year (2014–2018) estimates, U.S. Census Bureau. | N= 3,142 counties in 50 states and the District of Columbia with information  available on COVID-19 cases and deaths. Clinical studies show that it takes about 2–8 weeks  from onset to death in COVID-19 patients.  Mean (SD)  % Hispanic 9.3 (13.8)  % Black 9.9 (14.7) | 1- Center for Systems Science and Engineering (CSSE), Johns Hopkins University.  2- County Population Totals: 2010–2019, U.S. Census Bureau.  3- American Community Survey (ACS) 5-year (2014–2018) estimates, U.S. Census Bureau.  4- Area Health Resources File (AHRF) 2018–2019, Health Resources and Services Administration.  5- Kaiser Health News, the Kaiser Family Foundation | Multivariate linear regression models | Black ethnic residents;  Hispanic ethnic residents;  Uninsured residents;  Household size;  Household income (Median household income  ($1,000s) | Higher COVID-19 cases and deaths | - Counties with a higher proportion of the Black population and a higher proportion of adults with less than a high school diploma had disproportionately higher COVID-19 cases and deaths (β > 0, p<0.05 for all relations). - A higher proportion of the Hispanic population was associated with higher confirmed cases (β = 0.68, 95% CI = 0.48–0.87). - The correlation between the percentage of 25 years old and older adults with no high school andCOVID-19 cases (R = 0.32) and deaths (R = 0.37) was relatively strong and significant (P< 0.01). - There were positive relations between the percentage of Hispanic (B= 0.68; [0.48, 0.87]; p< .001) and Black (0.69 [0.53, 0.85]; p< .001) populations and COVID-19 cases per population unit. - Positive relations between the percentage of Hispanic (0.65 [0.1, 1.2]; p< .05) and Black population (1.57 [1.11, 2.03]; p< .001) and COVID-19 deaths were observed. - Higher median household income was associated with higher deaths per population unit 0.89(0.35, 1.42) p< .001. - There were no significant association between Average household size and % Uninsured population and confirmed COVID-19 deaths and confirmed COVID-19 cases. |
| 52 | (Weech-Maldonado et al., 2021) | US  all US nursing homes included in the CMS Nursing Home COVID-19 Public File, or 15,382 nursing homes, which mirrors the national census of facilities. | Cross-sectional study | - | N= 12,914 nursing home | CMS Nursing Home COVID-19 Public File as of October 25, 2020, Brown University’s LTCFocus, and Robert Graham Center’s Social Deprivation Index. | Multivariate regressions | Minority (not specified): categorizing | COVID-1 death | After controlling for interstate differences, facility-level resident characteristics, resource availability, and organizational characteristics, high-minority nursing homes had 61% more COVID-19 deaths as compared to nursing facilities with no minorities. |

References

Abedi, V., Olulana, O., Avula, V., Chaudhary, D., Khan, A., Shahjouei, S., . . . Zand, R. (2020). Racial, Economic, and Health Inequality and COVID-19 Infection in the United States. *J Racial Ethn Health Disparities*, 1-11. doi:10.1007/s40615-020-00833-4

Adegunsoye, A., Ventura, I. B., & Liarski, V. M. (2020). Association of Black Race with Outcomes in COVID-19 Disease: A Retrospective Cohort Study. *Annals of the American Thoracic Society, 17*(10), 1336-1339. doi:10.1513/AnnalsATS.202006-583RL

Adhikari, S., Pantaleo, N. P., Feldman, J. M., Ogedegbe, O., Thorpe, L., & Troxel, A. B. (2020). Assessment of Community-Level Disparities in Coronavirus Disease 2019 (COVID-19) Infections and Deaths in Large US Metropolitan Areas. *JAMA Network Open, 3*(7), e2016938-e2016938. doi:10.1001/jamanetworkopen.2020.16938

Ahmad, K., Erqou, S., Shah, N., Nazir, U., Morrison, A. R., Choudhary, G., & Wu, W. C. (2020). Association of poor housing conditions with COVID-19 incidence and mortality across US counties. *PLoS One, 15*(11), e0241327. doi:10.1371/journal.pone.0241327

Aldridge, R. W., Lewer, D., Katikireddi, S. V., Mathur, R., Pathak, N., Burns, R., . . . Hayward, A. (2020). Black, Asian and Minority Ethnic groups in England are at increased risk of death from COVID-19: indirect standardisation of NHS mortality data. *Wellcome Open Res, 5*, 88. doi:10.12688/wellcomeopenres.15922.2

Ayoubkhani, D., Nafilyan, V., White, C., Goldblatt, P., Gaughan, C., Blackwell, L., . . . Diamond, I. (2020). Ethnic-minority groups in England and Wales-factors associated with the size and timing of elevated COVID-19 mortality: a retrospective cohort study linking census and death records. *International journal of epidemiology*. doi:10.1093/ije/dyaa208

Azar, K. M. J., Shen, Z., Romanelli, R. J., Lockhart, S. H., Smits, K., Robinson, S., . . . Pressman, A. R. (2020). Disparities In Outcomes Among COVID-19 Patients In A Large Health Care System In California. *Health Aff (Millwood), 39*(7), 1253-1262. doi:10.1377/hlthaff.2020.00598

Baena-Diez, J. M., Barroso, M., Cordeiro-Coelho, S. I., Diaz, J. L., & Grau, M. (2020). Impact of COVID-19 outbreak by income: hitting hardest the most deprived. *Journal of Public Health, 42*(4), 698-703. doi:10.1093/pubmed/fdaa136

Baqui, P., Bica, I., Marra, V., Ercole, A., & van der Schaar, M. (2020). Ethnic and regional variations in hospital mortality from COVID-19 in Brazil: a cross-sectional observational study. *Lancet Glob Health, 8*(8), e1018-e1026. doi:10.1016/s2214-109x(20)30285-0

Boserup, B., McKenney, M., & Elkbuli, A. (2020). Disproportionate Impact of COVID-19 Pandemic on Racial and Ethnic Minorities. *American Surgeon, 86*(12), 1615-1622. doi:10.1177/0003134820973356

de Lusignan, S., Dorward, J., Correa, A., Jones, N., Akinyemi, O., Amirthalingam, G., . . . Zambon, M. (2020). Risk factors for SARS-CoV-2 among patients in the Oxford Royal College of General Practitioners Research and Surveillance Centre primary care network: a cross-sectional study. *Lancet Infectious Diseases, 20*(9), 1034-1042. doi:10.1016/S1473-3099(20)30371-6

de Lusignan, S., Joy, M., Oke, J., McGagh, D., Nicholson, B., Sheppard, J., . . . Tzortziou Brown, V. (2020). Disparities in the excess risk of mortality in the first wave of COVID-19: Cross sectional study of the English sentinel network. *Journal of Infection, 81*(5), 785-792. doi:10.1016/j.jinf.2020.08.037

DiMaggio, C., Klein, M., Berry, C., & Frangos, S. (2020). Black/African American Communities are at highest risk of COVID-19: spatial modeling of New York City ZIP Code-level testing results. *Annals of Epidemiology, 51*, 7-13. doi:10.1016/j.annepidem.2020.08.012

Drefahl, S., Wallace, M., Mussino, E., Aradhya, S., Kolk, M., Branden, M., . . . Andersson, G. (2020). A population-based cohort study of socio-demographic risk factors for COVID-19 deaths in Sweden. *Nature Communications, 11*(1). doi:10.1038/s41467-020-18926-3

Egede, L. E., Walker, R. J., Garacci, E., & Raymond, J. R., Sr. (2020). Racial/Ethnic Differences In COVID-19 Screening, Hospitalization, And Mortality In Southeast Wisconsin. *Health Aff (Millwood), 39*(11), 1926-1934. doi:10.1377/hlthaff.2020.01081

Farrell, R. J., O’Regan, R., O’Neill, E., Bowens, G., Maclellan, A., Gileece, A., . . . Burke, C. (2020). Sociodemographic variables as predictors of adverse outcome in SARS-CoV-2 infection: an Irish hospital experience. *Irish Journal of Medical Science*. doi:10.1007/s11845-020-02407-z

Fielding-Miller, R. K., Sundaram, M. E., & Brouwer, K. (2020). Social determinants of COVID-19 mortality at the county level. *PLoS One, 15*(10). doi:10.1371/journal.pone.0240151

Figueiredo, D. C. M. M., Gomes, L. B., Massuda, A., Gil-García, E., Vianna, R. P. T., & Daponte, A. (2020). Social determinants of health and COVID-19 infection in Brazil: an analysis of the pandemic. *Revista brasileira de enfermagem, 73*, e20200673. doi:10.1590/0034-7167-2020-0673

Goyal, M. K., Simpson, J. N., Boyle, M. D., Badolato, G. M., Delaney, M., McCarter, R., & Cora-Bramble, D. (2020). Racial and/or ethnic and socioeconomic disparities of SARS-CoV-2 infection among children. *Pediatrics, 146*(4).

Gu, T., Mack, J. A., Salvatore, M., Prabhu Sankar, S., Valley, T. S., Singh, K., . . . Mukherjee, B. (2020). Characteristics Associated With Racial/Ethnic Disparities in COVID-19 Outcomes in an Academic Health Care System. *JAMA network open, 3*(10), e2025197-e2025197. doi:10.1001/jamanetworkopen.2020.25197

Hawkins, D. (2020). Social Determinants of COVID-19 in Massachusetts, United States: An Ecological Study. *Journal of Preventive Medicine & Public Health, 53*(4), 220-227. doi:10.3961/jpmph.20.256

Hawkins, R. B., Charles, E. J., & Mehaffey, J. H. (2020). Socio-economic status and COVID-19–related cases and fatalities. *Public Health, 189*, 129-134. doi:10.1016/j.puhe.2020.09.016

Holmes, L., Jr., Enwere, M., Williams, J., Ogundele, B., Chavan, P., Piccoli, T., . . . Dabney, K. W. (2020). Black-White Risk Differentials in COVID-19 (SARS-COV2) Transmission, Mortality and Case Fatality in the United States: Translational Epidemiologic Perspective and Challenges. *Int J Environ Res Public Health, 17*(12). doi:10.3390/ijerph17124322

Holtgrave, D. R., Barranco, M. A., Tesoriero, J. M., Blog, D. S., & Rosenberg, E. S. (2020). Assessing racial and ethnic disparities using a COVID-19 outcomes continuum for New York State. *Annals of Epidemiology, 48*, 9-14. doi:10.1016/j.annepidem.2020.06.010

Hu, T., Yue, H., Wang, C., She, B., Ye, X., Liu, R., . . . Bao, S. (2020). Racial segregation, testing site access, and covid-19 incidence rate in Massachusetts, USA. *International Journal of Environmental Research and Public Health, 17*(24), 1-18. doi:10.3390/ijerph17249528

Ioannou, G. N., Locke, E., Green, P., Berry, K., O'Hare, A. M., Shah, J. A., . . . Fan, V. S. (2020). Risk Factors for Hospitalization, Mechanical Ventilation, or Death Among 10 131 US Veterans With SARS-CoV-2 Infection. *JAMA Network Open, 3*(9), e2022310-e2022310. doi:10.1001/jamanetworkopen.2020.22310

Joseph, N. P., Reid, N. J., Som, A., Li, M. D., Hyle, E. P., Dugdale, C. M., . . . Flores, E. J. (2020). Racial and ethnic disparities in disease severity on admission chest radiographs among patients admitted with confirmed coronavirus disease 2019: A retrospective cohort study. *Radiology, 297*(3), E303-E312. doi:10.1148/radiol.2020202602

Kabarriti, R., Brodin, N. P., Maron, M. I., Guha, C., Kalnicki, S., Garg, M. K., & Racine, A. D. (2020). Association of Race and Ethnicity With Comorbidities and Survival Among Patients With COVID-19 at an Urban Medical Center in New York. *JAMA Network Open, 3*(9), e2019795-e2019795. doi:10.1001/jamanetworkopen.2020.19795

Kaufman, H. W., Niles, J. K., & Nash, D. B. (2020). Disparities in SARS-CoV-2 Positivity Rates: Associations with Race and Ethnicity. *Popul Health Manag*. doi:10.1089/pop.2020.0163

Khan, K. S., Torpiano, G., McLellan, M., & Mahmud, S. (2020). The impact of socioeconomic status on 30-day mortality in hospitalized patients with COVID-19 infection. *J Med Virol*. doi:10.1002/jmv.26371

Khanijahani, A. (2021). Racial, ethnic, and socioeconomic disparities in confirmed COVID-19 cases and deaths in the United States: a county-level analysis as of November 2020. *Ethnicity & Health, 26*(1), 22-35. doi:10.1080/13557858.2020.1853067

Khanijahani, A., & Tomassoni, L. (2021). Socioeconomic and Racial Segregation and COVID-19: Concentrated Disadvantage and Black Concentration in Association with COVID-19 Deaths in the USA. *J Racial Ethn Health Disparities*. doi:10.1007/s40615-021-00965-1

Kim, H. N., Lan, K. F., Nkyekyer, E., Neme, S., Pierre-Louis, M., Chew, L., & Duber, H. C. (2020). Assessment of Disparities in COVID-19 Testing and Infection Across Language Groups in Seattle, Washington. *JAMA Network Open, 3*(9), e2021213-e2021213. doi:10.1001/jamanetworkopen.2020.21213

Lassale, C., Gaye, B., Hamer, M., Gale, C. R., & Batty, G. D. (2020). Ethnic disparities in hospitalisation for COVID-19 in England: The role of socioeconomic factors, mental health, and inflammatory and pro-inflammatory factors in a community-based cohort study. *Brain Behav Immun, 88*, 44-49. doi:10.1016/j.bbi.2020.05.074

Lieberman-Cribbin, W., Tuminello, S., Flores, R. M., & Taioli, E. (2020). Disparities in COVID-19 Testing and Positivity in New York City. *American Journal of Preventive Medicine, 59*(3), 326-332. doi:10.1016/j.amepre.2020.06.005

Loomba, R. S., Aggarwal, G., Aggarwal, S., Flores, S., Villarreal, E. G., Farias, J. S., & Lavie, C. J. (2021). Disparities in case frequency and mortality of coronavirus disease 2019 (COVID-19) among various states in the United States. *Ann Med, 53*(1), 151-159. doi:10.1080/07853890.2020.1840620

Madhav, K. C., Oral, E., Straif-Bourgeois, S., Rung, A. L., & Peters, E. S. (2020). The effect of area deprivation on COVID-19 risk in Louisiana. *PLoS One, 15*(12). doi:10.1371/journal.pone.0243028

Mahajan, U. V., & Larkins-Pettigrew, M. (2020). Racial demographics and COVID-19 confirmed cases and deaths: a correlational analysis of 2886 US counties. *Journal of Public Health, 42*(3), 445-447. doi:10.1093/pubmed/fdaa070

Misa, N. Y., Perez, B., Basham, K., Fisher-Hobson, E., Butler, B., King, K., . . . Anderson, E. S. (2020). Racial/ethnic disparities in COVID-19 disease burden & mortality among emergency department patients in a safety net health system. *Am J Emerg Med*. doi:10.1016/j.ajem.2020.09.053

Munoz-Price, L. S., Nattinger, A. B., Rivera, F., Hanson, R., Gmehlin, C. G., Perez, A., . . . Pezzin, L. E. (2020). Racial Disparities in Incidence and Outcomes Among Patients With COVID-19. *JAMA Network Open, 3*(9). doi:10.1001/jamanetworkopen.2020.21892

Niedzwiedz, C. L., O'Donnell, C. A., Jani, B. D., Demou, E., Ho, F. K., Celis-Morales, C., . . . Katikireddi, S. V. (2020). Ethnic and socioeconomic differences in SARS-CoV-2 infection: prospective cohort study using UK Biobank. *BMC Medicine, 18*(1), 1-14. doi:10.1186/s12916-020-01640-8

Ojinnaka, C. O., Adepoju, O. E., Burgess, A. V., & Woodard, L. (2020). Factors Associated with COVID-Related Mortality: the Case of Texas. *J Racial Ethn Health Disparities*, 1-6. doi:10.1007/s40615-020-00913-5

Ossimetha, A., Ossimetha, A., Kosar, C. M., & Rahman, M. (2021). Socioeconomic Disparities in Community Mobility Reduction and COVID-19 Growth. *Mayo Clinic Proceedings, 96*(1), 78-85.

Raine, S., Liu, A., Mintz, J., Wahood, W., Huntley, K., & Haffizulla, F. (2020). Racial and Ethnic Disparities in COVID-19 Outcomes: Social Determination of Health. *International Journal of Environmental Research and Public Health, 17*(21). doi:10.3390/ijerph17218115

Renelus, B. D., Khoury, N. C., Chandrasekaran, K., Bekele, E., Briggs, W. M., Ivanov, A., . . . Jamorabo, D. S. (2020). Racial Disparities in COVID-19 Hospitalization and In-hospital Mortality at the Height of the New York City Pandemic. *J Racial Ethn Health Disparities*, 1-7. doi:10.1007/s40615-020-00872-x

Rentsch, C. T., Kidwai-Khan, F., Tate, J. P., Park, L. S., King, J. T., Skanderson, M., . . . Justice, A. C. (2020). Patterns of COVID-19 testing and mortality by race and ethnicity among United States veterans: A nationwide cohort study. *PLoS Medicine, 17*(9). doi:10.1371/journal.pmed.1003379

Rodriguez, F., Solomon, N., de Lemos, J. A., Das, S. R., Morrow, D. A., Bradley, S. M., . . . Wang, T. Y. (2020). Racial and Ethnic Differences in Presentation and Outcomes for Patients Hospitalized with COVID-19: Findings from the American Heart Association's COVID-19 Cardiovascular Disease Registry. *Circulation*. doi:10.1161/circulationaha.120.052278

Sapey, E., Gallier, S., Mainey, C., Nightingale, P., McNulty, D., Crothers, H., . . . Ball, S. (2020). Ethnicity and risk of death in patients hospitalised for COVID-19 infection in the UK: an observational cohort study in an urban catchment area. *BMJ Open Respir Res, 7*(1). doi:10.1136/bmjresp-2020-000644

Soares, R. C. M., Mattos, L. R., & Raposo, L. M. (2020). Risk Factors for Hospitalization and Mortality due to COVID-19 in Espírito Santo State, Brazil. *Am J Trop Med Hyg, 103*(3), 1184-1190. doi:10.4269/ajtmh.20-0483

Weech-Maldonado, R., Lord, J., Davlyatov, G., Ghiasi, A., & Orewa, G. (2021). High-Minority Nursing Homes Disproportionately Affected by COVID-19 Deaths. *Frontiers in Public Health, 9*(246). doi:10.3389/fpubh.2021.606364

Yehia, B. R., Winegar, A., Fogel, R., Fakih, M., Ottenbacher, A., Jesser, C., . . . Cacchione, J. (2020). Association of Race With Mortality Among Patients Hospitalized With Coronavirus Disease 2019 (COVID-19) at 92 US Hospitals. *JAMA Netw Open, 3*(8), e2018039. doi:10.1001/jamanetworkopen.2020.18039

Zakeri, R., Bendayan, R., Ashworth, M., Bean, D. M., Dodhia, H., Durbaba, S., . . . Shah, A. M. (2020). A case-control and cohort study to determine the relationship between ethnic background and severe COVID-19. *EClinicalMedicine*, 100574. doi:10.1016/j.eclinm.2020.100574
