# Supplementary material for: A systematic review of racial/ethnic and socioeconomic disparities in COVID-19
Source: Int J Equity Health. 2021 Nov 24;20:248. doi: 10.1186/s12939-021-01582-4 (PMC8611382; doi:10.1186/s12939-021-01582-4)
Supplement: Supplementary file 3 — Additional file 3:. Assessment of methodological quality. [file 12939_2021_1582_MOESM3_ESM.pdf]

Additional file 3. Results of the methodological quality appraisal of the included studies.

Additional file 3. Results of reporting quality assessment of Racial/Ethnic and Socioeconomic Disparities in COVID-19 studies using JBI critical appraisal checklist for analytical cross-sectional studies										
**Author, year**	**Q1**	**Q2**	**Q3**	**Q4**	**Q5**	**Q6**	**Q7**	**Q8**	**Score (out of 8)**	**Score %**
Abedi et al. 2020. (1)	Y	Y	Y	Y	Y	Y	Y	Y	8	100
Adhikari et al. (2)	Y	U	Y	Y	N	N	Y	Y	5	62.5
Ahmad et al. 2020. (3)	Y	Y	Y	Y	Y	N	Y	Y	7	87.5
Baqui et al. 2020. (4)	Y	Y	Y	Y	N	N	Y	Y	6	75
de Lusignan et al. 2020. (5)	Y	Y	Y	Y	Y	Y	Y	Y	8	100
Egede et al. 2020. (6)	Y	Y	Y	Y	Y	Y	Y	Y	8	100
Farrell et al. 2020. (7)	Y	Y	Y	Y	Y	Y	Y	Y	8	100
Goyal et al. 2020. (8)	Y	Y	Y	Y	Y	U	Y	Y	7	87.5
Hawkins et al. 2020. (9)	Y	Y	Y	Y	N	N	Y	Y	6	75
Holmes et al. 2020. (10)	Y	N	Y	Y	N	N	Y	Y	5	62.5
Kim et al. 2020. (11)	Y	Y	Y	Y	N	N	Y	Y	6	62.5
Mahajan et al. 2020. (12)	Y	Y	Y	Y	N	N	Y	Y	6	62.5
Munoz-Price et al. 2020. (13)	Y	Y	Y	Y	Y	Y	Y	Y	8	100
Ojinnaka et al. 2020. (14)	Y	Y	Y	Y	Y	Y	Y	Y	8	100
Rodriguez et al. 2020. (15)	Y	Y	Y	Y	Y	Y	Y	Y	8	100
Lieberman-Cribbin et al. 2020. (16)	Y	Y	Y	Y	N	N	Y	Y	6	62.5
Loomba et al. 2021. (17)	Y	Y	Y	Y	Y	Y	Y	Y	8	100
Holtgrave et al. 2020. (18)	Y	Y	Y	Y	Y	N	Y	Y	7	87.5
Baena-Diez et al. 2020. (19)	Y	Y	Y	Y	Y	N	Y	Y	7	87.5
Boserup et al. 2020. (20)	Y	Y	Y	Y	Y	N	Y	Y	7	87.5
DiMaggio et al. 2020. (21)	Y	Y	Y	Y	Y	Y	Y	Y	8	100
Fielding-Miller et al. 2020. (22)	Y	Y	Y	Y	Y	Y	Y	Y	8	100
Figueiredo et al. 2020. (23)	Y	Y	Y	Y	Y	Y	Y	Y	8	100
Hu et al. 2020. (24)	Y	Y	Y	Y	N	N	Y	Y	6	62.5
Madhav et al. 2020. (25)	Y	Y	Y	Y	N	N	Y	Y	6	62.5
Raine et al. 2020. (26)	Y	Y	Y	Y	Y	Y	Y	Y	8	100
Ossimetha et al. 2021. (27)	Y	Y	Y	Y	N	N	Y	Y	6	62.5
Khanijahani et al. 2021. (28)	Y	Y	Y	Y	Y	Y	Y	Y	8	100
Khanijahani et al. 2021. (29)	Y	Y	Y	Y	Y	Y	Y	Y	8	100
Weech-Maldonado et al. 2021. (30)	Y	Y	Y	Y	Y	Y	Y	Y	8	100
**Average score**										**86.25**

Y: Yes N: No U: Unclear NA: Not Applicable
Q1: Were the criteria for inclusion in the sample clearly defined?
Q2: Were the study subjects and the setting described in detail?
Q3: Was the exposure measured in a valid and reliable way?
Q4: Were objective, standard criteria used for measurement of the condition?
Q5: Were confounding factors identified?
Q6: Were strategies to deal with confounding factors stated?
Q7: Were the outcomes measured in a valid and reliable way?
Q8: Was appropriate statistical analysis used?

Additional file 3: Results of reporting quality assessment of Racial/Ethnic and Socioeconomic Disparities in COVID-19studies using JBI critical appraisal checklist for cohort studies													
**Author, year**	**Q1**	**Q2**	**Q3**	**Q4**	**Q5**	**Q6**	**Q7**	**Q8**	**Q9**	**Q10**	**Q11**	**Score (out of 11)**	**Score %**
Adegunsoye et al. 2020. (31)	Y	Y	Y	U	Y	Y	Y	Y	Y	NA	Y	9	81.8
Aldridge et al. 2020. (32)	Y	Y	Y	N	N	Y	Y	Y	Y	NA	Y	8	72.7
Azar et al. 2020. (33)	Y	Y	Y	Y	Y	Y	Y	Y	Y	NA	Y	10	90.9
Drefahl et al. 2020. (34)	Y	Y	Y	Y	Y	Y	Y	Y	Y	Y	Y	11	100
Gu et al. 2020. (35)	N	Y	Y	Y	Y	Y	Y	Y	Y	NA	Y	9	81.8
Ioannou et al. 2020. (36)	Y	Y	Y	Y	N	Y	Y	Y	Y	Y	Y	10	90.9
Joseph et al. 2020. (37)	Y	Y	Y	Y	Y	Y	Y	Y	Y	Y	Y	11	100
Kabarriti et al. 2020. (38)	Y	Y	Y	Y	N	Y	Y	Y	Y	Y	Y	10	90.9
Kaufman et al. 2020. (39)	Y	Y	Y	Y	Y	Y	Y	Y	Y	Y	Y	11	100
Khan et al. 2020. (40)	Y	Y	Y	Y	Y	Y	Y	Y	Y	Y	Y	11	100
Lassale et al. 2020. (41)	Y	Y	Y	Y	Y	Y	Y	Y	Y	Y	Y	11	100
Misa et al. 2020. (42)	Y	Y	Y	Y	Y	Y	Y	Y	Y	NA	Y	10	90.9
Niedzwiedz et al. 2020. (43)	Y	Y	Y	Y	Y	Y	Y	Y	Y	N	Y	10	90.9
Renelus et al. 2020. (44)	Y	Y	Y	U	U	Y	Y	Y	Y	Y	Y	9	81.8
Rentsch et al. 2020. (45)	Y	Y	Y	Y	Y	Y	Y	Y	Y	NA	Y	10	90.9
Sapey et al. 2020. (46)	Y	Y	Y	Y	Y	Y	Y	Y	Y	NA	Y	10	90.9
Soares et al. 2020. (47)	Y	Y	Y	Y	Y	Y	Y	Y	Y	NA	Y	10	90.9
Yehia et al. 2020. (48)	Y	Y	Y	Y	Y	Y	Y	Y	Y	NA	Y	10	90.9
Ali et al. 2021. (49)	Y	Y	Y	Y	Y	Y	Y	Y	Y	NA	Y	10	90.9
Ayoubkhani et al. 2020. (50)	Y	Y	Y	Y	Y	Y	Y	Y	Y	NA	Y	10	90.9
Hawkins et al. 2020. (51)	Y	Y	Y	Y	Y	Y	Y	Y	Y	NA	Y	10	90.9
**Average score**													**90.9**

Y: Yes N: No U: Unclear NA: Not Applicable

Q1: Were the two groups similar and recruited from the same population?
Q2: Were the exposures measured similarly to assign people to both exposed and unexposed groups?
Q3: Was the exposure measured in a valid and reliable way?
Q4: Were confounding factors identified?
Q5: Were strategies to deal with confounding factors stated?
Q6: Were the groups/participants free of the outcome at the start of the study (or at the moment of exposure)?
Q7: Were the outcomes measured in a valid and reliable way?
Q8: Was the follow up time reported and sufficient to be long enough for outcomes to occur?
Q9: Was follow up complete, and if not, were the reasons to loss to follow up described and explored?
Q10: Were strategies to address incomplete follow up utilized?
Q11: Was appropriate statistical analysis used?

Additional file3. Results of reporting quality assessment of Racial/Ethnic and Socioeconomic Disparities in COVID-19 studies using JBI critical appraisal checklist for case control studies												
Author, year	**Q1**	**Q2**	**Q3**	**Q4**	**Q5**	**Q6**	**Q7**	**Q8**	**Q9**	**Q10**	**Score (out of 10)**	**Score %**
Zakeri et al. 2020. (52)	Y	Y	Y	Y	Y	Y	Y	Y	Y	Y	10	100
**Average score**												**100**

Y: Yes N: No U: Unclear NA: Not Applicable
Q1: Were the groups comparable other than the presence of disease in cases or the absence of disease in controls?
Q2: Were cases and controls matched appropriately?
Q3: Were the same criteria used for identification of cases and controls?
Q4: Was exposure measured in a standard, valid and reliable way?
Q5: Was exposure measured in the same way for cases and controls?
Q6: Were confounding factors identified?
Q7: Were strategies to deal with confounding factors stated?
Q8: Were outcomes assessed in a standard, valid and reliable way for cases and controls?
Q9: Was the exposure period of interest long enough to be meaningful?
Q10: Was appropriate statistical analysis used?

References

1. Abedi V, Olulana O, Avula V, Chaudhary D, Khan A, Shahjouei S, et al. Racial, Economic, and Health Inequality and COVID-19 Infection in the United States. Journal of racial and ethnic health disparities. 2020:1-11.
2. Adhikari S, Pantaleo NP, Feldman JM, Ogedegbe O, Thorpe L, Troxel AB. Assessment of Community-Level Disparities in Coronavirus Disease 2019 (COVID-19) Infections and Deaths in Large US Metropolitan Areas. JAMA Network Open. 2020;3(7):e2016938-e.
3. Ahmad K, Erqou S, Shah N, Nazir U, Morrison AR, Choudhary G, et al. Association of poor housing conditions with COVID-19 incidence and mortality across US counties. PloS one. 2020;15(11):e0241327.
4. Baqui P, Bica I, Marra V, Ercole A, van der Schaar M. Ethnic and regional variations in hospital mortality from COVID-19 in Brazil: a cross-sectional observational study. The Lancet Global health. 2020;8(8):e1018-e26.
5. de Lusignan S, Dorward J, Correa A, Jones N, Akinyemi O, Amirthalingam G, et al. Risk factors for SARS-CoV-2 among patients in the Oxford Royal College of General Practitioners Research and Surveillance Centre primary care network: a cross-sectional study. Lancet Infectious Diseases. 2020;20(9):1034-42.
6. Egede LE, Walker RJ, Garacci E, Raymond JR, Sr. Racial/Ethnic Differences In COVID-19 Screening, Hospitalization, And Mortality In Southeast Wisconsin. Health affairs (Project Hope). 2020;39(11):1926-34.
7. Farrell RJ, O'Regan R, O'Neill E, Bowens G, Maclellan A, Gileece A, et al. Sociodemographic variables as predictors of adverse outcome in SARS-CoV-2 infection: an Irish hospital experience. Irish Journal of Medical Science. 2020.
8. Goyal MK, Simpson JN, Boyle MD, Badolato GM, Delaney M, McCarter R, et al. Racial and/or ethnic and socioeconomic disparities of SARS-CoV-2 infection among children. Pediatrics. 2020;146(4).
9. Hawkins D. Social Determinants of COVID-19 in Massachusetts, United States: An Ecological Study. Journal of Preventive Medicine & Public Health. 2020;53(4):220-7.
10. Holmes L, Jr., Enwere M, Williams J, Ogundele B, Chavan P, Piccoli T, et al. Black-White Risk Differentials in COVID-19 (SARS-COV2) Transmission, Mortality and Case Fatality in the United

States: Translational Epidemiologic Perspective and Challenges. Int J Environ Res Public Health. 2020;17(12).
11. Kim HN, Lan KF, Nkyekyer E, Neme S, Pierre-Louis M, Chew L, et al. Assessment of Disparities in COVID-19 Testing and Infection Across Language Groups in Seattle, Washington. JAMA Network Open. 2020;3(9):e2021213-e.
12. Mahajan UV, Larkins-Pettigrew M. Racial demographics and COVID-19 confirmed cases and deaths: a correlational analysis of 2886 US counties. Journal of Public Health. 2020;42(3):445-7.
13. Munoz-Price LS, Nattinger AB, Rivera F, Hanson R, Gmehlin CG, Perez A, et al. Racial Disparities in Incidence and Outcomes Among Patients With COVID-19. Jama Network Open. 2020;3(9).
14. Ojinnaka CO, Adepoju OE, Burgess AV, Woodard L. Factors Associated with COVID-Related Mortality: the Case of Texas. Journal of racial and ethnic health disparities. 2020:1-6.
15. Rodriguez F, Solomon N, de Lemos JA, Das SR, Morrow DA, Bradley SM, et al. Racial and Ethnic Differences in Presentation and Outcomes for Patients Hospitalized with COVID-19: Findings from the American Heart Association's COVID-19 Cardiovascular Disease Registry. Circulation. 2020.
16. Lieberman-Cribbin W, Tuminello S, Flores RM, Taioli E. Disparities in COVID-19 Testing and Positivity in New York City. American Journal of Preventive Medicine. 2020;59(3):326-32.
17. Loomba RS, Aggarwal G, Aggarwal S, Flores S, Villarreal EG, Farias JS, et al. Disparities in case frequency and mortality of coronavirus disease 2019 (COVID-19) among various states in the United States. Annals of medicine. 2021;53(1):151-9.
18. Holtgrave DR, Barranco MA, Tesoriero JM, Blog DS, Rosenberg ES. Assessing racial and ethnic disparities using a COVID-19 outcomes continuum for New York State. Annals of Epidemiology. 2020;48:9-14.
19. Baena-Diez JM, Barroso M, Cordeiro-Coelho SI, Diaz JL, Grau M. Impact of COVID-19 outbreak by income: hitting hardest the most deprived. Journal of Public Health. 2020;42(4):698-703.
20. Boserup B, McKenney M, Elkbuli A. Disproportionate Impact of COVID-19 Pandemic on Racial and Ethnic Minorities. American Surgeon. 2020;86(12):1615-22.
21. DiMaggio C, Klein M, Berry C, Frangos S. Black/African American Communities are at highest risk of COVID-19: spatial modeling of New York City ZIP Code-level testing results. Annals of Epidemiology. 2020;51:7-13.
22. Fielding-Miller RK, Sundaram ME, Brouwer K. Social determinants of COVID-19 mortality at the county level. Plos One. 2020;15(10).
23. Figueiredo DCMM, Gomes LB, Massuda A, Gil-García E, Vianna RPT, Daponte A. Social determinants of health and COVID-19 infection in Brazil: an analysis of the pandemic. Revista brasileira de enfermagem. 2020;73:e20200673.
24. Hu T, Yue H, Wang C, She B, Ye X, Liu R, et al. Racial segregation, testing site access, and covid-19 incidence rate in Massachusetts, USA. International Journal of Environmental Research and Public Health. 2020;17(24):1-18.
25. Madhav KC, Oral E, Straif-Bourgeois S, Rung AL, Peters ES. The effect of area deprivation on COVID-19 risk in Louisiana. Plos One. 2020;15(12).
26. Raine S, Liu A, Mintz J, Wahood W, Huntley K, Haffizulla F. Racial and Ethnic Disparities in COVID-19 Outcomes: Social Determination of Health. International Journal of Environmental Research and Public Health. 2020;17(21).
27. Ossimetha A, Ossimetha A, Kosar CM, Rahman M. Socioeconomic Disparities in Community Mobility Reduction and COVID-19 Growth. Mayo Clinic Proceedings. 2021;96(1):78-85.
28. Khanijahani A, Tomassoni L. Socioeconomic and Racial Segregation and COVID-19: Concentrated Disadvantage and Black Concentration in Association with COVID-19 Deaths in the USA. Journal of racial and ethnic health disparities. 2021.

29. Khanijahani A. Racial, ethnic, and socioeconomic disparities in confirmed COVID-19 cases and deaths in the United States: a county-level analysis as of November 2020. Ethnicity & Health. 2021;26(1):22-35.
30. Weech-Maldonado R, Lord J, Davlyatov G, Ghiasi A, Orewa G. High-Minority Nursing Homes Disproportionately Affected by COVID-19 Deaths. Frontiers in Public Health. 2021;9(246).
31. Adegunsoye A, Ventura IB, Liarski VM. Association of Black Race with Outcomes in COVID-19 Disease: A Retrospective Cohort Study. Annals of the American Thoracic Society. 2020;17(10):1336-9.
32. Aldridge RW, Lewer D, Katikireddi SV, Mathur R, Pathak N, Burns R, et al. Black, Asian and Minority Ethnic groups in England are at increased risk of death from COVID-19: indirect standardisation of NHS mortality data. Wellcome open research. 2020;5:88.
33. Azar KMJ, Shen Z, Romanelli RJ, Lockhart SH, Smits K, Robinson S, et al. Disparities In Outcomes Among COVID-19 Patients In A Large Health Care System In California. Health affairs (Project Hope). 2020;39(7):1253-62.
34. Drefahl S, Wallace M, Mussino E, Aradhya S, Kolk M, Branden M, et al. A population-based cohort study of socio-demographic risk factors for COVID-19 deaths in Sweden. Nature Communications. 2020;11(1).
35. Gu T, Mack JA, Salvatore M, Prabhu Sankar S, Valley TS, Singh K, et al. Characteristics Associated With Racial/Ethnic Disparities in COVID-19 Outcomes in an Academic Health Care System. JAMA Network Open. 2020;3(10):e2025197-e.
36. Ioannou GN, Locke E, Green P, Berry K, O'Hare AM, Shah JA, et al. Risk Factors for Hospitalization, Mechanical Ventilation, or Death Among 10 131 US Veterans With SARS-CoV-2 Infection. JAMA Network Open. 2020;3(9):e2022310-e.
37. Joseph NP, Reid NJ, Som A, Li MD, Hyle EP, Dugdale CM, et al. Racial and ethnic disparities in disease severity on admission chest radiographs among patients admitted with confirmed coronavirus disease 2019: A retrospective cohort study. Radiology. 2020;297(3):E303-E12.
38. Kabarriti R, Brodin NP, Maron MI, Guha C, Kalnicki S, Garg MK, et al. Association of Race and Ethnicity With Comorbidities and Survival Among Patients With COVID-19 at an Urban Medical Center in New York. JAMA Network Open. 2020;3(9):e2019795-e.
39. Kaufman HW, Niles JK, Nash DB. Disparities in SARS-CoV-2 Positivity Rates: Associations with Race and Ethnicity. Population health management. 2020.
40. Khan KS, Torpiano G, McLellan M, Mahmud S. The impact of socioeconomic status on 30-day mortality in hospitalized patients with COVID-19 infection. Journal of medical virology. 2020.
41. Lassale C, Gaye B, Hamer M, Gale CR, Batty GD. Ethnic disparities in hospitalisation for COVID-19 in England: The role of socioeconomic factors, mental health, and inflammatory and pro-inflammatory factors in a community-based cohort study. Brain, behavior, and immunity. 2020;88:44-9.
42. Misa NY, Perez B, Basham K, Fisher-Hobson E, Butler B, King K, et al. Racial/ethnic disparities in COVID-19 disease burden & mortality among emergency department patients in a safety net health system. The American journal of emergency medicine. 2020.
43. Niedzwiedz CL, O'Donnell CA, Jani BD, Demou E, Ho FK, Celis-Morales C, et al. Ethnic and socioeconomic differences in SARS-CoV-2 infection: prospective cohort study using UK Biobank. BMC Medicine. 2020;18(1):1-14.
44. Renelus BD, Khoury NC, Chandrasekaran K, Bekele E, Briggs WM, Ivanov A, et al. Racial Disparities in COVID-19 Hospitalization and In-hospital Mortality at the Height of the New York City Pandemic. Journal of racial and ethnic health disparities. 2020:1-7.
45. Rentsch CT, Kidwai-Khan F, Tate JP, Park LS, King JT, Skanderson M, et al. Patterns of COVID-19 testing and mortality by race and ethnicity among United States veterans: A nationwide cohort study. PLoS Medicine. 2020;17(9).

46. Sapey E, Gallier S, Mainey C, Nightingale P, McNulty D, Crothers H, et al. Ethnicity and risk of death in patients hospitalised for COVID-19 infection in the UK: an observational cohort study in an urban catchment area. BMJ open respiratory research. 2020;7(1).
47. Soares RCM, Mattos LR, Raposo LM. Risk Factors for Hospitalization and Mortality due to COVID-19 in Espírito Santo State, Brazil. The American journal of tropical medicine and hygiene. 2020;103(3):1184-90.
48. Yehia BR, Winegar A, Fogel R, Fakih M, Ottenbacher A, Jesser C, et al. Association of Race With Mortality Among Patients Hospitalized With Coronavirus Disease 2019 (COVID-19) at 92 US Hospitals. JAMA network open. 2020;3(8):e2018039.
49. Ali H, Alshukry A, Marafie SK, AlRukhayes M, Ali Y, Abbas MB, et al. Outcomes of COVID-19: Disparities by ethnicity. Infection, Genetics and Evolution. 2021;87:104639.
50. Ayoubkhani D, Nafilyan V, White C, Goldblatt P, Gaughan C, Blackwell L, et al. Ethnic-minority groups in England and Wales-factors associated with the size and timing of elevated COVID-19 mortality: a retrospective cohort study linking census and death records. International journal of epidemiology. 2020.
51. Hawkins RB, Charles EJ, Mehaffey JH. Socio-economic status and COVID-19–related cases and fatalities. Public Health. 2020;189:129-34.
52. Zakeri R, Bendayan R, Ashworth M, Bean DM, Dodhia H, Durbaba S, et al. A case-control and cohort study to determine the relationship between ethnic background and severe COVID-19. EClinicalMedicine. 2020:100574.
